# Excitation-wavelength-dependent persistent luminescence from single-component nonstoichiometric CaGa_x_O_4_:Bi for dynamic anti-counterfeiting

**DOI:** 10.1038/s41377-024-01635-7

**Published:** 2024-10-10

**Authors:** Bo-Mei Liu, Yue Lin, Yingchun Liu, Bibo Lou, Chong-Geng Ma, Hui Zhang, Jing Wang

**Affiliations:** 1 School of Chemical Engineering and Light Industry, Guangdong University of Technology, Guangzhou, China; 2 Ministry of Education Key Laboratory of Bioinorganic and Synthetic Chemistry, State Key Laboratory of Optoelectronic Materials and Technologies, School of Chemistry, Sun Yat-sen University, Guangzhou, China; 3 Guangdong Laboratory of Chemistry and Fine Chemical Industry Jieyang Center, Jieyang, China; 4 School of Optoelectronic Engineering & CQUPT-BUL Innovation Institute, Chongqing University of Posts and Telecommunications, Chongqing, China; 5 Nanchang Research Institute, Sun Yat-sen University, Nanchang, Jiangxi China

**Keywords:** Metamaterials, Imaging and sensing

## Abstract

Materials capable of dynamic persistent luminescence (PersL) within the visible spectrum are highly sought after for applications in display, biosensing, and information security. However, PersL materials with eye-detectable and excitation-wavelength-dependent characteristics are rarely achieved. Herein, a nonstoichiometric compound CaGa_x_O_4_:Bi (*x* < 2) is present, which demonstrates ultra-long, color-tunable PersL. The persistent emission wavelength can be tuned by varying the excitation wavelength, enabling dynamic color modulation from the green to the orange region within the visible spectrum. Theoretical calculations, in conjunction with experimental observations, are utilized to elucidate the thermodynamic charge transitions of various defect states, thereby providing insights into the relationship between Bi^3+^ emitters, traps, and multicolored PersL. Furthermore, the utility of color-tunable PersL materials and flexible devices is showcased for use in visual sensing of invisible ultraviolet light, multicolor display, information encryption, and anti-counterfeiting. These discoveries create new opportunities to develop smart photoelectric materials with dynamically controlled PersL for various applications.

## Abstract (web summary)

A nonstoichiometric CaGa_x_O_4_:Bi (*x* < 2) design introduces compositional defects that generate various traps near Bi ions, enabling excitation-wavelength-dependent persistent luminescence (PersL) and holding promise for advanced anti-counterfeiting applications.

## Graphical abstract

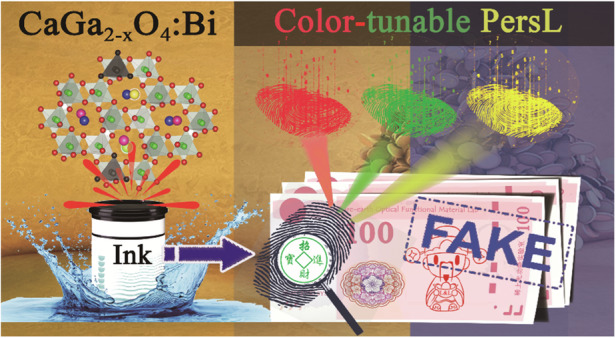

## Introduction

Counterfeiting poses a pervasive threat to industries worldwide, impacting areas ranging from politics and the economy to military operations and daily life^1^. To combat counterfeiting, various anti-counterfeiting strategies have been explored, encompassing holograms, watermarks, radiofrequency identification, barcodes, two-dimensional codes, and luminescence labels^2^. Among these anti-counterfeiting strategies, luminescence anti-counterfeiting has attracted increasing attention due to its excellent optical properties of luminescent materials, including high emission intensity, multiple emission colors, long emission lifetime, and various emission modes such as photoluminescence, chemiluminescence, and mechanoluminescence. Currently, the most mature luminescence anti-counterfeiting technology relies on luminescence anti-counterfeiting labels, which are extensively employed in currencies worldwide^3–6^. However, in the face of rampant counterfeiting, conventional low-level luminescence anti-counterfeiting labels alone are inadequate to meet the escalating demands for anti-counterfeiting measures.

Low-level anti-counterfeiting label is often the result of conventional luminescent materials with the typical traits of unicolor, unitemporal, and unimodal emission. The immutability of conventional anti-counterfeiting labels, where the information cannot be changed after encoding, is a disadvantage for advanced anti-counterfeiting requiring flexible adjustability. Consequently, the pursuit of materials capable of multicolor, multitemporal, and multimodal luminescence emerges as a promising solution for addressing the challenges of advanced anti-counterfeiting measures^7,8^.

Recently, novel luminescent materials have been developed that exhibit dynamic multicolor persistence luminescence (PersL) with time dependence and allow information storage in the time-space dimension to enable advanced dynamic encryption^9–11^. One notable property, PersL, has intrigued researchers since its discovery, owing to its time-dependent emission^12,13^. Another aspect, dynamic multicolor luminescence, empowers luminescent materials with superior performance in various applications^14,15^. Examples include organics^16,17^, carbonaceous materials^18,19^, metal-organic frameworks^11,20^, and organic-inorganic hybrids^21^, which can emit multicolor luminescence under different excitation conditions by modulating the composition, crystallinity, phase, and dopant of the material. In 2019, Huang et al. developed several organic phosphors capable of color-tunable (380–505 nm) and ultra-long (2.45 s) phosphorescence, achieved through triplet state stabilization^16^. Since then, organic room temperature phosphorescence systems have been extensively studied for their potential application in information encryption, optical sensing, and multiplexed bioassays^9,22^. Although above carbonaceous or organic systems show remarkable color-tunable PersL, their poor stability and inferior PersL (usually lasting only a few seconds and invisible to the naked eye) significantly limit their potential applications^23–25^. In theory, inorganic multicolor PersL materials with greater stability and superior luminescence hold greater practical potential. However, nearly all inorganic multicolor PersL has been achieved through altering the doping of luminescent centers or adjusting their concentrations in multi-component materials^26–28^. To the best of our knowledge, researchers have not yet found an inorganic single-component material with eye-detectable excitation-wavelength-dependent PersL.

In contrast to traditional luminescent labels that store information in static form and lack support for color or time-dependent display, multicolor PersL luminescence can offer dynamic encryption. However, it also requires careful encoding through time and additional external physical stimuli (e.g. thermal, mechanical, chemical, or photostimulated^23,29^) to exhibit labels in a time-dependent manner with dynamic color tuning. For instance, photostimulated luminescence (PSL), a luminescence response to optical stimuli, can accelerate the release of stored energy in PersL materials, resulting in rapid changes in emitting colors or intensity^30^. With their smart photo-switchable properties, PSL materials hold potential applications in the fields of information storage and anti-counterfeiting. Therefore, it can be envisioned that the synergistic integration of multicolor, PersL, and PSL functionalities in a single-component can encrypt multilevel authenticating information for advanced anti-counterfeiting, effectively increasing the counterfeiting threshold.

Herein, the unprecedented integration of multicolor (orange-yellow-green), time-dependent (PersL), and two-modal (excitation-dependent PL and PSL) emissions in inorganic single-component CaGa_x_O_4_:Bi (*x* < 2) via nonstoichiometric component design is demonstrated. The formation energies and corresponding charge-transition levels of various defects and defect complexes in a nonstoichiometric model system are investigated by a combination of calculations and experiments, revealing that the introduction of Ga vacancy facilitates the creation of five different trap states for color-tunable PersL. By ingeniously combining the PSL of CaGa_x_O_4_:Bi with its distinct excitation-wavelength-dependent PersL, a multilevel anti-counterfeiting technology is developed and presented. This comprehensive multicolor PersL material has potential applications for ultraviolet detection, full-color displays, information encryption, and anti-counterfeiting.

## Results

### Photophysical properties of nonstoichiometric phase

**Fig. 1:**
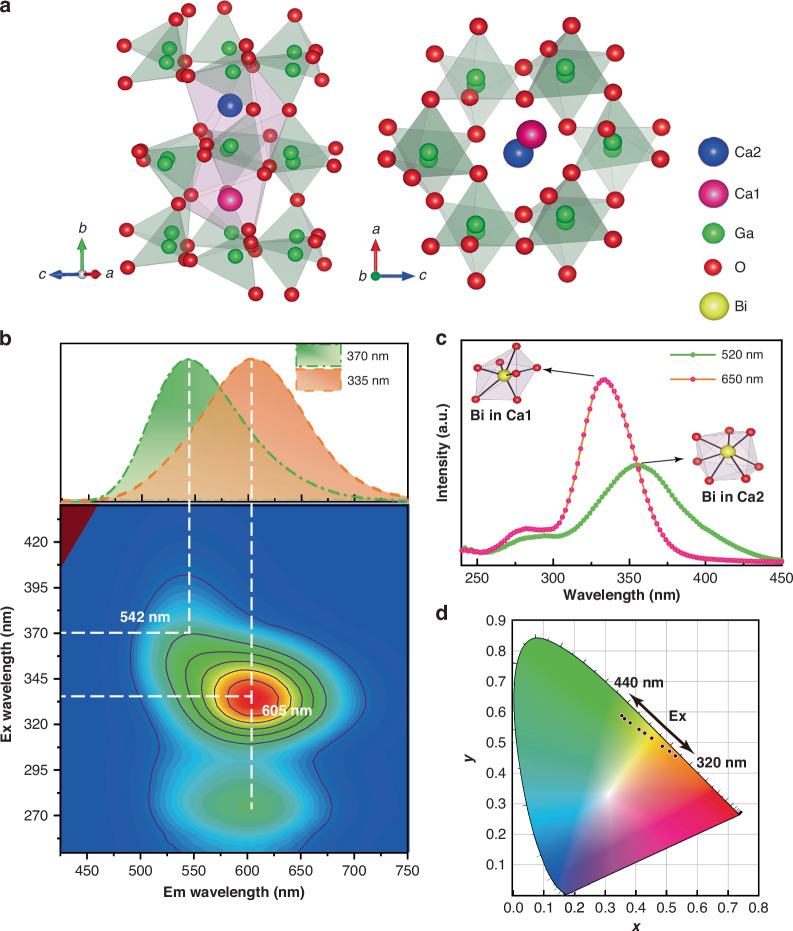
Photoluminescence and structural characterization of CaGa_1.97_O_4_:0.5%Bi under ambient conditions. **a** Crystal structure of CaGa_2_O_4_ viewed along a different axis. **b** Excitation–emission mapping of CaGa_1_._97_O_4_:0.5%Bi. The upper inset shows the PL spectra of the sample after excitation at 335 nm and 370 nm, respectively. **c** Excitation spectra of the sample at 520 (green line) and 650 nm (pink line). Inset: Bi ions located at the Ca1 and Ca2 sites. **d** Trajectory of the color modulation, recorded by changing the excitation from 320 to 440 nm, depicted in the CIE coordinate diagram

To validate our scheme, a series of CaGa_x_O_4_:0.5%Bi (1.95 ≤ *x* ≤ 2.01) samples were synthesized via the solid-state route. All these samples exhibit an orthorhombic structure with space group *Pnam*, and the designed nonstoichiometric components can not disrupt the long-range order of the crystal lattice of CaGa_2_O_4_ (Fig. 1a, S1). Energy-dispersive spectroscopy of the Ca, Ga, O, and Bi contents shows that the elements and the induced Ga vacancies (V_Ga_) are uniformly distributed in the matrix (Figure S2). Moreover, the Raman patterns show no signs of superstructure compared with stoichiometric CaGa_2_O_4_:Bi, implying that vacancies are randomly distributed in the CaGa_x_O_4_:Bi (1.97 ≤ *x* < 2), without disrupting the long-range crystalline order (Figure S3). Absorption analysis demonstrates that V_Ga_ significantly affects the Bi microenvironment in the Ga-deficient phase, enhancing the light absorption activity of the Bi ions (Figure S4a).

Herein, CaGa_x_O_4_:Bi (1.95 ≤ *x* ≤ 2.01) exhibits distinct excitation-wavelength-dependent (Figs. 1b, d, S5, S6). Increasing V_Ga_ content significantly enhances the emission intensity of Bi^3+^ and leads to a new Bi^3+^-O^2-^ charge transfer excitation band at 275 nm, possibly due to a local environmental distortion caused by V_Ga_. The emission intensity of Ga-deficient CaGa_1.97_O_4_:0.5%Bi is approximately six times stronger than CaGa_2_O_4_:0.5%Bi (Figure S7). The increase in PL emission intensity might result from the impact of nonstoichiometric composition defects on Bi centers.

Next, the nonstoichiometric strategy is demonstrated using Ga-deficient CaGa_1.97_O_4_:0.5%Bi. As depicted in Fig. 1b, the green (542 nm) and orange (605 nm) emission of CaGa_1.97_O_4_:0.5%Bi originate from the electronic transition of Bi^3+^ pairs to an intervalence charge transfer. The excitation band at 335 and 355 nm are initially ascribed to Bi^3+^(6 *s*^2^*)*,Bi^3+^(6*s*^2^*)* → Bi^4+^(6*s)*,Bi^2+^(6*s*^2^6*p)* with two types of Bi^3+^ ions (denoted as ${{\rm{Bi}}}_{{\rm{Ca}}1}$ and ${{\rm{Bi}}}_{{\rm{Ca}}2}$) by substituting the Ca1 and Ca2 sites, respectively (Fig. 1a, c, Table S1). The structural and electronic properties of the ground and excited states for the two types of Bi^3+^-related transition processes will be fully discussed later.

The ratiometric change in emission intensities of two types of Bi^3+^-related emitters, as a function of the excitation wavelength, leads to color-tunable luminescence. With the excitation wavelength ranging from 320 to 420 nm, the emission peak exhibits a noticeable blueshift from orange to green along with a variation of the main peak from 605 to 542 nm. Resolved PL spectra are simultaneously depicted in Figure S8. The color variations of CaGa_1.97_O_4_:0.5%Bi in response to different excitation wavelengths are shown in the Commission International de l’Eclairage (CIE) coordinate diagram in Fig. 1d.

**Fig. 2:**
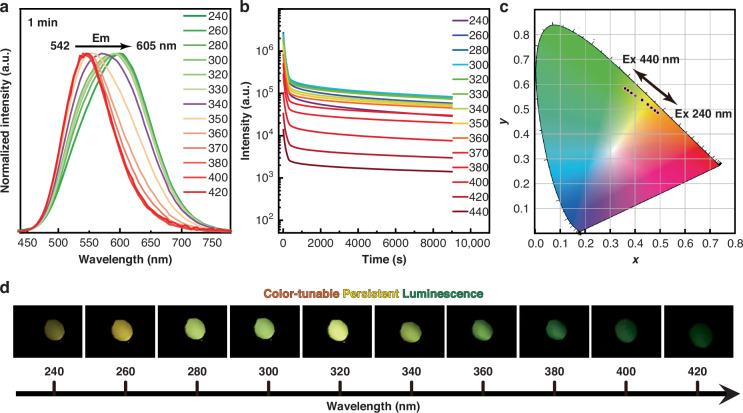
PersL properties of CaGa_1.97_O_4_:0.5%Bi^3+^ under ambient conditions. **a** Normalized excitation-wavelength-dependent PersL spectra of CaGa_1.97_O_4_:0.5%Bi (irradiation time, 5 min; interval, 1 min). **b** Persistent decay monitored at the corresponding wavelength according to Figure S11 after 5 min of Xe lamp excitation from 240 to 440 nm. **c** The trajectory of tunable PersL colors recorded when changing the excitation wavelength from 240 to 440 nm, depicted in the CIE coordinate diagram. **d** UV color chart showing the ability to visually detect specific wavelengths in the UV range using CaGa_1.97_O_4_:0.5%Bi

Interestingly, the PersL ranging from 605 to 542 nm emitted by the Ga-deficient phase can be observed with the naked eye after removing the UV light (ranging from 240 to 420 nm) (Fig. 2a, Movie S1). And the excitation-wavelength-dependent emission can be maintained after different UV excitations (Figure S9). In contrast, no PersL can be observed with the naked eye in the stoichiometric CaGa_2_O_4_:0.5%Bi phase. Analysis of the PersL spectra reveals that the tunable color from green to orange of Ga-deficient CaGa_1.97_O_4_:0.5%Bi remains unchanged for over 15 min following excitation from 240 to 420 nm (Fig. 2a, S10, S11). At different excitation wavelengths, the corresponding emission decay curves in the 240–380 nm range are very close to each other, and the intensities vary within an order of magnitude (Fig. 2b). The specific wavelength of UV light can be accurately determined by correlating the excitation wavelength with the CIE chromaticity coordinates (Fig. 2c). Visual PersL images could be captured by the naked eye when the invisible UV excitation changes from 240 to 420 nm (Fig. 2d), demonstrating the potential utility for UV light detection. Additionally, it is noteworthy that the Ga-deficient CaGa_1.97_O_4_:Bi can be effectively activated by direct sunlight (Figure S12).

### Mechanism investigations of multicolor PersL

To gain further insight into the unique optical properties, the samples were further analyzed by thermoluminescence (TL). Increasing V_Ga_ concentration leads to increased TL intensity and new TL peaks (Figure S13), suggesting that there is a highly dense trapping level originating from V_Ga_ or related defects. The irradiation-time-dependent and interval-time-dependent TL curves of CaGa_1.97_O_4_:Bi show that more than one type of trap is involved in the PersL process (Figure S14). The above results clearly show that the nonstoichiometric CaGa_x_O_4_:Bi (*x* < 2) has superb capabilities in energy collection, storage, conversion, and colorful PersL.

**Fig. 3:**
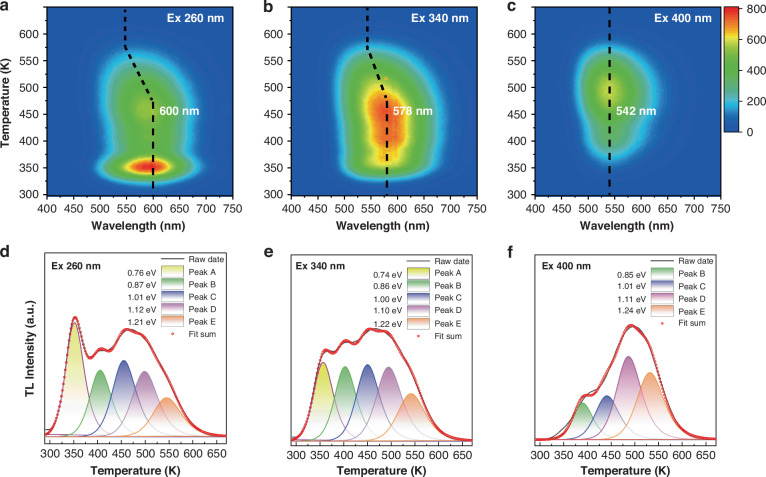
TL properties of CaGa_1.97_O_4_:0.5%Bi^3+^ under different excitation wavelengths. The 3D-TL spectra of sample pre-irradiated with light at **a** 260 nm, **b** 340 nm, and **c** 400 nm for 5 min. Decomposed 2D-TL spectra of the sample after 5 min of excitation with **d** 260 nm, **e** 340 nm, and **f** 400 nm light

Later, the effectiveness of different excitation wavelengths for colorful PersL was verified by TL measurements. Figure 3a–f shows the 3D- and 2D-TL curves of Ga-deficient CaGa_1.97_O_4_:0.5%Bi irradiated with 260, 340, and 400 nm light, respectively. Interestingly, different excitation wavelengths also lead to different TL emissions, which coincide with the wavelength of PersL (Fig. 3a–c). As shown in Fig. 3d–f, the 2D-TL curve is a combination of five traps (A-E: ~0.75, ~0.86, ~1.01, ~1.11, ~1.22 eV) theoretically fitted by a general-order TL kinetic expression (Equation S1), suggesting that the traps are distributed over a wide range of depths. The detailed trap parameters are listed in Table S2. In comparison, the shallow trap A/B/C releases the emission light at 600 nm with increasing temperature (Fig. 3a, d). Once the temperature exceeds 475 K, D/E starts to release stored energy, resulting in a shift of the emission light to 542 nm (Fig. 3a–c). This result suggests that the trapped electrons or holes may reside in two isolated Bi^3+^ ions corresponding to the two assemblies (A/B/C and B/C/D/E) of in-gap trap states. Most of these shallow traps are dominant for emission at 600 nm, as they have quite small formation energy to match the excited state of an isolated Bi^3+^ center. The remaining deeper traps, mainly for 542 nm emission, correspond to another isolated Bi^3+^ center. The details are described below. There are many possibilities for defects or defect complexes in this type of nonstoichiometric system with V_Ga_ and V_O_. Based on all these results, it is posit that the defects associated with different Bi emitters could act as carrier trapping centers with diverse depths, making CaGa_1.97_O_4_:0.5%Bi a novel excitation‐wavelength-dependent PersL.

**Fig. 4:**
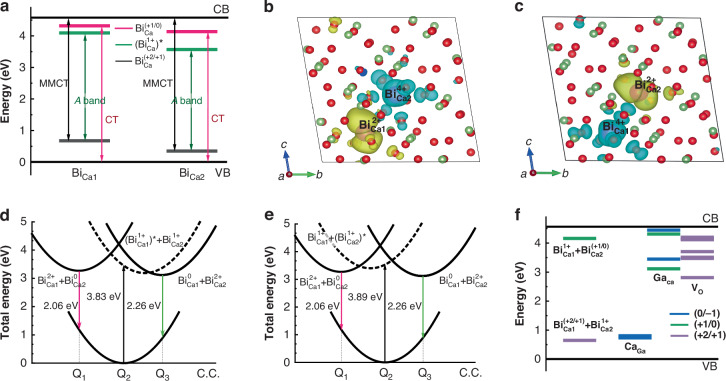
Dynamic process of the excited states in PersL. **a** Transition level position and the zero phonon transition energies of the isolated Bi^3+^ ions in Ca1 or Ca2 sites. Charge density scheme of the Bi^3+^ pair excited state of ${{\rm{Bi}}}_{{\rm{Ca}}1}^{2+}-{{\rm{Bi}}}_{{\rm{Ca}}2}^{4+}$ (**b**) and ${{\rm{Bi}}}_{{\rm{Ca}}1}^{4+}-{{\rm{Bi}}}_{{\rm{Ca}}2}^{2+}$ (**c**). Configurational coordinate diagrams of the CaGa_2_O_4_:Bi^3+^ potential surfaces as a function of the generalized configuration coordinate for the emission processes caused by the $6{{\rm{s}}}^{2}\to 6{\rm{s}}6{\rm{p}}$ excitation of Bi^3+^ in Ca1 (**d**) and Ca2 (**e**) sites. (**f**) Depth of the trap centers provided by ${{\rm{Ca}}}_{{\rm{Ga}}}$, ${{\rm{Ga}}}_{{\rm{Ca}}}$ and ${{\rm{V}}}_{{\rm{O}}}$, as well as the transition levels provided by the emission centers, Bi^3+^ pairs

To provide greater insight into the underlying microscopic mechanism of multicolor PersL formation in nonstoichiometric CaGa_x_O_4_:Bi (*x* < 2), the trapping and de-trapping processes in CaGa_2_O_4_:Bi^3+^ are elucidated through first-principles calculations. The calculation methods are provided in Supplementary Information. First, the emission processes of bismuth as an activator are investigated. Previous studies have indicated that the existence of varying stable charge states for Bi in various oxides, ranging from +2 to +4, poses a challenge in studies of recombination processes^31^. For Bi^3+^ ions, there are charge transfer transitions, including ${{\rm{Bi}}}^{3+}\to \,{{\rm{Bi}}}^{4+}+{e}_{{\rm{CBM}}}$ and ${{\rm{Bi}}}^{3+}+{h}_{{\rm{VBM}}}\to \,{{\rm{Bi}}}^{2+}$ observed in experiment, expect for the $6{{\rm{s}}}^{2}\to 6{\rm{s}}6{\rm{p}}$ transition inter Bi^3+^ ions. Furthermore, the charge transfer transitions between two nearby Bi^3+^ pairs also have been observed and studied^32^. In CaGa_2_O_4_:Bi^3+^, the transition level diagram and the zero phonon transition energies of the isolated Bi ions in Ca1 site (denoted as ${{\rm{Bi}}}_{{\rm{Ca}}1}$) and Ca2 site (${{\rm{Bi}}}_{{\rm{Ca}}2}$) are provided in Fig. 4a, where the relatively higher transition levels of ${{\rm{Bi}}}_{{\rm{Ca}}1}$ site is caused by the shorter ligand bond length and the stronger nephelauxetic effect. The ${{\rm{Bi}}}_{{\rm{Ca}}1}$ defect can act as a stable hole trap, whereas the ${{\rm{Bi}}}_{{\rm{Ca}}1}$ defect can be a stable electron trap. The calculated *A* band excitation ($6{{\rm{s}}}^{2}\to 6{\rm{s}}6{\rm{p}}$) of ${{\rm{Bi}}}_{{\rm{Ca}}1}^{3+}$ is 3.83 eV, slightly smaller than the 3.89 eV of ${{\rm{Bi}}}_{{\rm{Ca}}2}^{3+}$, which can assign the peaking energies of the two observed excitation spectra. However, no corresponding emission of the isolated Bi ions can match well with the measurements (Table S1), as the electron on ${{\rm{Bi}}}_{{\rm{Ca}}1}^{3+}$-6p orbital and the hole on ${{\rm{Bi}}}_{{\rm{Ca}}2}^{3+}$-6s orbital are both too close to the band edge and the isolated Bi ions are not expected to be good emission centers.

According to the calculated binding energies (${E}_{{\rm{b}}}=-0.21{\rm{eV}}$) with the expression of ${E}_{{\rm{B}}}={E}^{{\rm{f}}}\left[{{\rm{Bi}}}^{3+}{\rm{pair}}\right]-2\times {E}^{{\rm{f}}}[{{\rm{Bi}}}^{3+}]$, there is a tendency to form Bi^3+^ pairs in CaGa_2_O_4_, meanwhile, it can be stable in the excited state of ${{\rm{Bi}}}_{{\rm{Ca}}1}^{4+}-{{\rm{Bi}}}_{{\rm{Ca}}2}^{2+}$ (Fig. 4b) and ${{\rm{Bi}}}_{{\rm{Ca}}1}^{2+}-{{\rm{Bi}}}_{{\rm{Ca}}2}^{4+}$ (Fig. 4c), which corresponds to the charge transfer transitions from ${{\rm{Bi}}}_{{\rm{Ca}}1}^{3+}$ to ${{\rm{Bi}}}_{{\rm{Ca}}2}^{3+}$ and that form ${{\rm{Bi}}}_{{\rm{Ca}}2}^{3+}$ to ${{\rm{Bi}}}_{{\rm{Ca}}1}^{3+}$, being similar to the case in GdAlO_3_^32^. The calculated energies of the emissions origin from the two excited states of Bi pairs are 2.06 eV (${{\rm{Bi}}}_{{\rm{Ca}}1}^{4+}-{{\rm{Bi}}}_{{\rm{Ca}}2}^{2+}$) and 2.26 eV (${{\rm{Bi}}}_{{\rm{Ca}}1}^{2+}-{{\rm{Bi}}}_{{\rm{Ca}}2}^{4+}$), in great agreement with the observation. Based on the calculations of the equilibrium geometric structures of the ground and the mentioned excited states, as well as their relative energy positions compared to the ground state, the configurational coordinate diagram was constructed to depict the geometric relaxations and emission processes. The *A* band excitation of ${{\rm{Bi}}}_{{\rm{Ca}}1}^{3+}$ results in the expansion of the local structure at Ca1 site (Table S1) and it tends to relax to the lower excited state of ${{\rm{Bi}}}_{{\rm{Ca}}1}^{2+}-{{\rm{Bi}}}_{{\rm{Ca}}2}^{4+}$ with closer geometric configuration as shown in Fig. 4d. Similarly, there is the Bi^3+^ pair emission from the ${{\rm{Bi}}}_{{\rm{Ca}}1}^{4+}-{{\rm{Bi}}}_{{\rm{Ca}}2}^{2+}$ state that is induced by the *A* band excitation of ${{\rm{Bi}}}_{{\rm{Ca}}2}^{3+}$ (Fig. 4e).

Understanding the species of defects is essential for comprehending the PersL mechanism. Therefore, the formation energies of the trap centers were calculated with PBE functional considering the Ga-poor environment (Figure S15). The fermi level is around 1 eV in the pristine host and the dominative intrinsic defects are Ca_Ga_ acting as hole traps and Ga_Ca_, V_O_ acting as electron traps. With the substituting of Bi^3+^ ions at the Ca^2+^ sites, the concentration of Ca_Ga_ defects increases and the fermi level position rises. The trap depths of the trap\emission centers are calculated in hybrid DFT calculation for a more accurate description of their energy positions relative to the band edges and the results are plotted in Fig. 4f. All these results unambiguously indicate that the introduction of Ga/O vacancies in nonstoichiometric CaGa_x_O_4_:Bi (*x* < 2) results in the appearance of a series of in-gap defect levels with different depths. Two Ga_Ca_ and three V_O_ defects are perfectly matched with the A-E trap depth around 1 eV according to the TL fitting results (Fig. 3d–f). For convenience, the case is considered that the PersL of Bi^3+^ pair doped CaGa_2_O_4_ is caused by the interband excitation first. In this condition, the electron and hole are released by the host band absorption and partially stored in the trap centers provided by the intrinsic defects. Meanwhile, the Bi^3+^-pair can also capture the released charge carriers, which results in the change of the charge state of the emission centers and the formation of the ${{\rm{Bi}}}_{{\rm{Ca}}1}^{4+}-{{\rm{Bi}}}_{{\rm{Ca}}2}^{3+}$ and ${{\rm{Bi}}}_{{\rm{Ca}}1}^{3+}-{{\rm{Bi}}}_{{\rm{Ca}}2}^{2+}$ before the de-trapping processes (Fig. 4f). The trap depths of transition level of the Bi^3+^ pair in Fig. 4f show that the trapped electron or hole is mostly localized in an isolated Bi^3+^ ion. After the de-trapping processes, the Bi pairs in the ${{\rm{Bi}}}_{{\rm{Ca}}1}^{4+}-{{\rm{Bi}}}_{{\rm{Ca}}2}^{3+}$ charge state will subsequently capture a released electron from the trap centers and recombine with the hole that is mostly localized in ${{\rm{Bi}}}_{{\rm{Ca}}1}^{4+}$, which will relax from the excited state of ${\left({{\rm{Bi}}}_{{\rm{Ca}}1}^{3+}\right)}^{\star }-{{\rm{Bi}}}_{{\rm{Ca}}2}^{3+}$ to ${{\rm{Bi}}}_{{\rm{Ca}}1}^{2+}-{{\rm{Bi}}}_{{\rm{Ca}}2}^{4+}$ and provide the emission of 2.26 eV (Fig. 4d). Parallelly, there is charge carriers capture and geometric relaxation happened for Bi pairs in the ${{\rm{Bi}}}_{{\rm{Ca}}1}^{4+}-{{\rm{Bi}}}_{{\rm{Ca}}2}^{3+}$ charge state and provide the emission of 2.02 eV. With the radiation of energies above 3.89 eV, the isolated Bi ions in two Ca sites both can be excited and provide the released electron and hole. However, the quantity of hole is much less than the interband excitation, which is exhibited in the variation of the wavelength in PersL emission. As for the radiation at about 3.83 eV, the excitation can only active the ${\rm{B}}{{\rm{i}}}_{{\rm{Ca}}1}^{3+}$ and release the charge carrier of electrons. In the recombination processes, it mainly provides the emission of 2.26 eV. In short, the different excitation energies can result in the forming of different types of charge carriers and different charge states of the emission centers, which results in different recombination processes and the variation of emission wavelength.

**Fig. 5:**
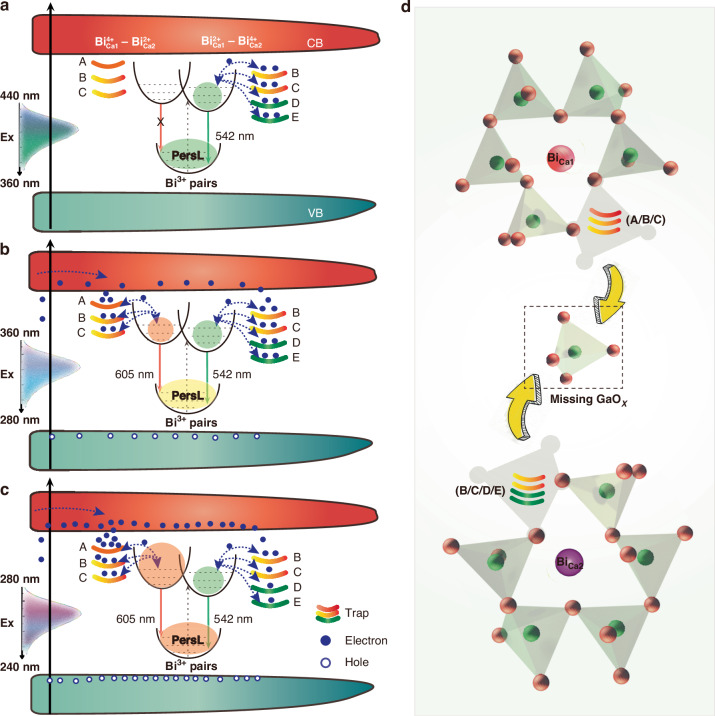
Rational design of excitation-wavelength-dependent PersL in the CaGa_1.97_O_4_:0.5%Bi lattice. Schematic representation of the color-tunable PersL mechanism under the excitation located in (**a**) 240–280 nm (orange PersL), (**b**) 280–360 nm (yellow PersL), (**c**) 360–440 nm (green PersL), respectively. **d** The microenvironment and nearby traps surrounding ${\rm{B}}{{\rm{i}}}_{{\rm{Ca}}1}$ and ${\rm{B}}{{\rm{i}}}_{{\rm{Ca}}2}$ after de-intercalation of GaO_x_ (*x* < 4) unit

According to experimental PersL color observations, excitation light is roughly categorized into three bands (Fig. 2c): ①360–440 nm, resulting in green emission; ②280–360 nm, resulting in yellow emission; ③240–280 nm, resulting in orange emission. Based on experimental observations and theoretical calculations, a plausible mechanism for the multicolor PersL is proposed, as schematically depicted in Fig. 5.

Upon excitation at 400 nm, the B/C/D/E traps store energy and subsequently release only 542 nm light as the temperature rises (Fig. 3c, f). Since the light at 400 nm (3.1 eV < Eg) can only be absorbed by isolated ${\rm{B}}{{\rm{i}}}_{{\rm{Ca}}1}^{3+}$ (Fig. 1c, S4b), implying that these traps and stored energy are confined in the vicinity of ${\rm{B}}{{\rm{i}}}_{{\rm{Ca}}1}^{3+}$. A similar situation occurs under 360–440 nm excitation, as shown in Fig. 5a. After 400 nm irradiation ceases, in the beginning, the electrons can migrate directly to nearby Bi^3+^-pair by tunneling, followed by transfer to the ${{\rm{Bi}}}_{{\rm{Ca}}1}^{2+}-{{\rm{Bi}}}_{{\rm{Ca}}2}^{4+}$ ions and provide the emission of 542 nm through the tunneling channels.

Upon excitation at 340 nm, PersL is centered at 578 nm, representing an intermediate state between the conduction band (CB)-dominant (<280 nm) and isolated Bi^3+^-absorption-dominant (> 360 nm) (Fig. 3b, e). As shown in Fig. 5b, increasing the excitation wavelength from 280 to 360 nm reduces the electrons jumping through CB to the shallow traps, with most electrons localizing in isolated Bi^3+^. The ratiometric change of the filled traps, in conjunction with two neighboring excited states of the Bi-pairs, leads to a tunable PersL emission ranging from 542–600 nm.

Upon excitation at 260 nm, both the host and the two isolated Bi^3+^ ions can absorb the energy of UV light, with corresponding traps harvesting energy from CB and the isolated Bi^3+^ ions (Fig. 5c). Initially after UV irradiation interruption, direct recombination between the electrons released from trap A and the excited state of the ${{\rm{Bi}}}_{{\rm{Ca}}1}^{4+}-{{\rm{Bi}}}_{{\rm{Ca}}2}^{3+}$ pair predominates the PersL process. Because the energy can hardly transfer to and release from B/C/D/E traps (> 0.86 eV) due to the larger gap between the defect level and CB than A trap (0.75 eV). The higher absorption capacity and release rate (evidenced by the extremely intense TL band of trap A; Figure S14) of the microenvironment consisting of Bi^3+^ pairs and trap A leads to orange PersL emission. The above calculation results have fully supported this point.

The effective activation of multicolor PersL by different light reveals that the energy collection, storage, and conversion within the microenvironment consists of defects and Bi^3+^ pairs. The introduced Bi^3+^ ions and V_Ga_ exert significant influence on PersL, while the symbiotic defects such as Ca_Ga_, Ga_Ca_, V_O_ are likely generated to maintain charge balance. These traps may settle in the vicinity of Bi^3+^ ions, altering the microenvironment of the Bi emitters from that within the interconnected six-membered-ring of GaO_4_ tetrahedra to a five-membered-ring configuration (Fig. 5d). Meanwhile, no evidence of electron or hole release was determined by EPR analysis (Figure S16). It is therefore concluded that the carriers released during excitation in the PersL process are electron−hole pairs^33^, which support PersL by simultaneously separating into electrons at Ga_Ca_, V_O,_ and holes in Ca_Ga_.

### Multicolor PersL for anti-counterfeiting applications

**Fig. 6:**
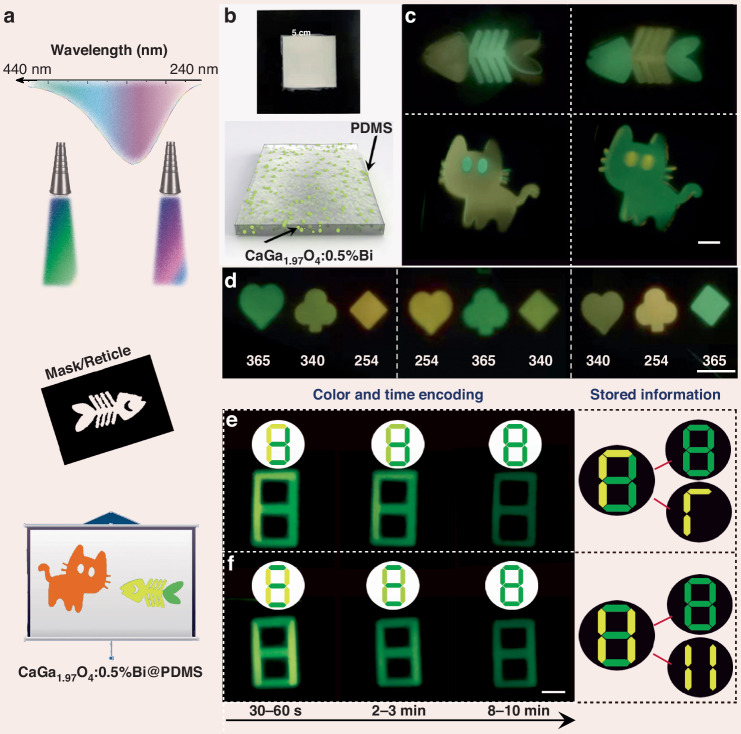
Demonstration of the excitation-wavelength-dependent PersL for multicolor display and optical information storage. **a** Schematic diagram of the experimental setup for multicolor display by varying the excitation wavelength from 240 to 440 nm. **b** Schematic diagram and optical image of the composite film produced. **c** PersL photographs of colorful fish and cat patterns recorded on the composite film. Note that the green and yellow parts were recorded after switching off the 365 and 254 nm hand-held lighter, respectively. **d** Poker patterns of hearts, clubs, and diamonds were obtained simultaneously by randomly arranging the 254/340/365 nm light from the Xe lamp through the mask. **e**, **f** The images are color- and time-dependent PersL images of figure “8” pre-irradiated with a 254 nm (yellow part, 10 s) and 365 nm (green part, 3 min) hand-held lighter. Note that the top images in the white circle are the simulation patterns according to the PersL images; the patterns on the right are the stored information. Scale bar: 1 cm

Inspired by the unique PersL properties of CaGa_1_._97_O_4_:0.5%Bi, two authentication experiments were designed to demonstrate the multicolor PersL performance for photonic displays and information encryption. Different patterns, such as a fish, a cat, and a digit, with different sizes from 1 to 5 cm were fabricated by mask/reticle with the pattern present only on a portion of the final exposed area (Fig. 6a). Figure 6b shows the optical image and schematic of the fabricated composite film, where the CaGa_1_._97_O_4_:0.5%Bi powder was dispersed in a soft PDMS matrix.

The first authentication pathway relies on multicolor PersL, which is generated in response to different excitation wavelength stimuli. When the excitation wavelength falls within the range of 240–420 nm, control PersL imaging of the film showed colorful patterns including orange, yellow, and green (Fig. 6c, d). Notably, the unique feature of this demonstration is that the multicolor patterns can be easily write-in by tuning the UV excitation light. The second pathway involves creating a new matrix of time- and color-coded patterns by selectively irradiating different areas of the film. Both the decay time and PersL color can be easily distinguished with the naked eye (Fig. 6e, f). For instance, Fig. 6f shows that a mixed PersL image (digit “8”) is overlaid by two overlapping images (yellow “11” and green “8”) printed on the corresponding pattern by pre-irradiation with 254 nm and 365 nm light, respectively. Then, the colorful PersL image transitions from a mixed yellow “11” and green “8” (0–3 min) to a single green “8” (8–10 min). Furthermore, with this approach, the window duration of the yellow and green PersL can be adjusted by varying the UV light irradiation time. This outcome lays the groundwork for a novel information encryption method based on the excitation wavelength, emission colors, and decay time of PersL. Additionally, the outstanding virtue of our method is that the multicolor time-dependent codes can be easily write-in by tuning the UV excitation light and visually read-out by the naked eye.

**Fig. 7:**
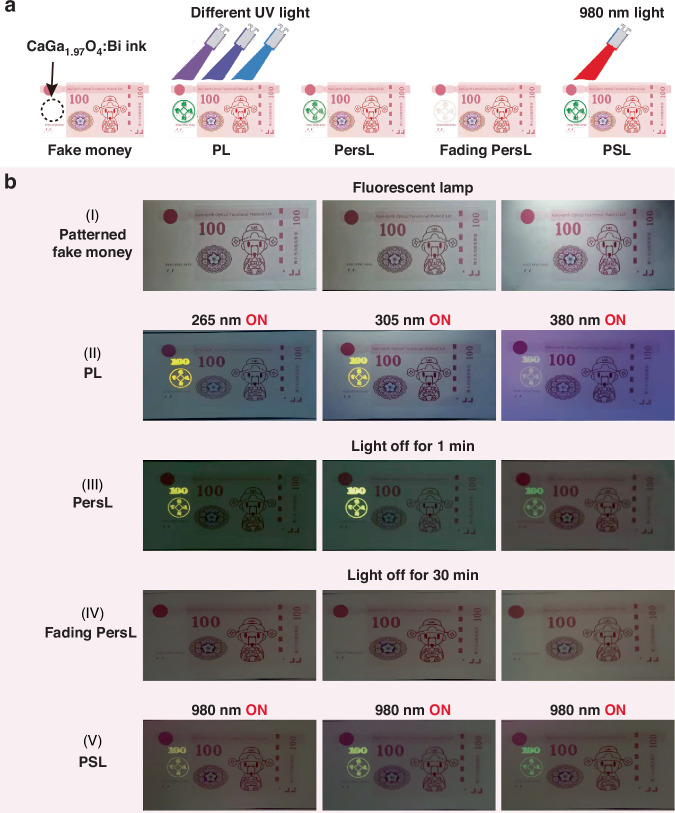
Potential anti-counterfeiting applications based on CaGa_1.97_O_4_:0.5%Bi^3+^ ink. **a** Design and concept of dynamic anti-counterfeiting based on three-color PL, PersL, and PSL. **b** Dynamic encryption and authentication process using the color-tunable CaGa_1_._97_O_4_:0.5%Bi^3+^ ink. (I) photo of self-designed banknote. (II) Orange-yellow-green patterned images were captured with excitation at 265 nm, 305 nm, and 380 nm, respectively. After removing the excitation for (III) 1 min and (IV) 30 min. (V) 980 nm light was applied to the labeled pattern

To employ this material directly in an advanced anti-counterfeiting situation, the CaGa_1_._97_O_4_:0.5%Bi ink was printed onto the self-designed banknote (fake money). As shown in Fig. 7a, b–I, the printed information (e.g. a digit pattern “100” and Chinese characters “招财进宝”) on the banknote is nearly invisible under ambient light. To verify the authenticity of the banknote, the security pattern (“100” and “招财进宝”) was irradiated with three different UV lights (265 nm, 305 nm, and 380 nm). If the security pattern can be observed on the banknote in the three kinds of PL and PersL mode (265 nm/orange, 305 nm/yellow, and 380 nm/green) (Fig. 7b-II, -III), it is deemed authentic; otherwise, it is considered counterfeit. Interestingly, the stored orange/yellow/green light can be read again with 980 nm light after PersL has completely disappeared (Fig. 7b-IV, -V). Compared to other fluorescent inks such as NIR ink (green fluorescent), UV ink (yellow fluorescent), and UV ink (green fluorescent), this color-tunable ink offers a heightened level of security for protecting the authenticity of various important and valuable items, such as documents, stamps, tickets, etc (Figure S17).

Furthermore, the CaGa_1_._97_O_4_:0.5%Bi embedded PDMS matrix as an optical data storage medium is notable for its high-security level. As shown in Movie S1, S2, the digital video demonstrates the dynamic PersL and PSL of the bee logo. Using a silk-screen printing technique, a “bee” is encoded in the composite film. The encrypted pattern, devoid of any discernible information, can only be deciphered under specific conditions: exposure to UV light (265, 305, 365, 380 nm LEDs) or NIR light (980 nm LED). The “bee” pattern can be read visually in dark-field PL, PersL, and PSL mode.

## Discussion

In summary, the nonstoichiometric strategy has proven effective in creating excitation-wavelength-dependent PersL materials CaGa_x_O_4_:Bi (*x* < 2). Integration of experimental observations and theoretical calculations reveals that Ga vacancies introduced by nonstoichiometric design lead to diverse microenvironments consisting of Bi^3+^ pairs and related traps. The alteration of Bi coordination environment from the six-membered rings of GaO_4_ tetrahedra in stoichiometric CaGa_2_O_4_:Bi to the Ga-deficient five-membered rings in the nonstoichiometric CaGa_x_O_4_:Bi (*x* < 2) accounts for the color-tunable PersL. Importantly, a novel strategy was devised to achieve the modulation and differential display of multicolor PersL by combining excitation wavelength, irradiation time, and PSL. Leveraging the unique features of multicolored PersL in CaGa_x_O_4_:Bi, an advanced luminescence anti-counterfeiting method was developed for the first time. Results demonstrate that the designed luminescence anti-counterfeiting strategy based on the color-tunable PersL is sufficiently secure and more user-friendly. Consequently, these findings will unlock new opportunities for designing highly integrated smart luminescence materials and drive their innovative applications in information encryption, multilevel anti-counterfeiting, and photonic displays.

## Materials and methods

### Synthesis of Bi-doped stoichiometric and nonstoichiometric calcium gallate

The solid-state reaction technique has been applied for the synthesis of stoichiometric and nonstoichiometric calcium gallate. Products with nominal compositions of Ca_0.995_Bi_0.005_Ga_x_O_4_ (*x* = 2.01, 2.00, 1.99, 1.97, and 1.95) were designed and synthesized, where the nominal deficiency relative to stoichiometric were 0.5% Ca deficiency (*x* = 2.01), 0.5% Ga deficiency (*x* = 1.99), 1.5% Ga deficiency (*x* = 1.97) and 2.5% Ga deficiency (*x* = 1.95), respectively. The raw materials were dried in a box furnace at 400 °C for 24 h to eliminate moisture effects. The required amounts of the high-purity starting materials CaCO_3_ (Aladdin,99.99%), Ga_2_O_3_ (Aladdin, 99.99%), and Bi_2_O_3_ (Aladdin, 99.99%) were blended and heated twice at 1000 °C for a period of 6 h in a covered alumina crucible. Also, undoped and 1% Bi-doped stoichiometric and nonstoichiometric calcium gallate samples were prepared in the same way as a reference.

### Preparation of CaGa_1.97_O_4_:0.5%Bi@PDMS composites

PDMS (Sylgard 184, Dow Corning) was employed as the elastic matrix to provide shape and support for colorful PersL powders. First, 2.5 g of PDMS base resin and 0.5 g of curing agent were mixed in a square mold with a side of length 50 mm. Then, 1 g of CaGa_1.97_O_4_:0.5%Bi powders were dispersed in the above mixture by mechanical stirring for 10 min. After curing at 80 °C for 3 h, the PDMS-based composites were obtained.

### Preparation of CaGa_1.97_O_4_:0.5%Bi ink

The luminescent label, comprising a polymer matrix embedded with the synthesized colorful PersL powders, is utilized to generate patterns for colorful PersL. The luminescent labels on banknotes and paper are produced using silk-screen printing techniques. Additionally, common fluorescent ink, NIR ink (green fluorescent), UV ink (yellow fluorescent), and UV ink (green fluorescent) are also purchased for comparison.

### Characterization and calculations section

The details are in Supplementary Information.

## Supplementary information

Supplementary information

Annotation for movie 1–2

Dynamic PersL of the luminescent logo

NIR irradiation PSL of the luminescent logo

## Data Availability

All data in the paper are present in the main text and/or the Supplementary Materials, which will also be provided by the corresponding author upon reasonable request.
