# Supplementary material for: Excitation-wavelength-dependent persistent luminescence from single-component nonstoichiometric CaGaxO4:Bi for dynamic anti-counterfeiting
Source: Light Sci Appl. 2024 Oct 10;13:286. doi: 10.1038/s41377-024-01635-7 (PMC11467341; doi:10.1038/s41377-024-01635-7)
Supplement: Supplementary file 1 — Supplementary information [file 41377_2024_1635_MOESM1_ESM.docx]

**Supplementary Information for**

**Excitation-wavelength-dependent persistent luminescence from single-component nonstoichiometric CaGa_x_O_4_:Bi for dynamic anti-counterfeiting**

Bo-Mei Liu^1,2,3*^, Yue Lin^1^, Yingchun Liu^1^, Bibo Lou^4^, Chong-Geng Ma^4*^, Hui Zhang^1^, and Jing Wang^2,5*^

*^1^ School of Chemical Engineering and Light Industry, Guangdong University of Technology, Guangzhou 510006, China*

*^2^ Ministry of Education Key Laboratory of Bioinorganic and Synthetic Chemistry, State Key Laboratory of Optoelectronic Materials and Technologies,* *School of Chemistry, Sun Yat-sen University, Guangzhou 510006, China*

*^3^ Guangdong Laboratory of Chemistry and Fine Chemical Industry Jieyang Center, Jieyang 515200, China*

*^4^ School of Optoelectronic Engineering & CQUPT-BUL Innovation Institute, Chongqing University of Posts and Telecommunications, 400065 Chongqing, China*

*^5^ Nanchang Research Institute, Sun Yat-sen University, Nanchang, Jiangxi, 330099, China*

** Corresponding authors.*

*E-mail address:* [*liubomei@gdut.edu.cn*](mailto:liubomei@gdut.edu.cn) (B.-M. Liu); cgma.ustc@gmail.com (C.-G Ma); [ceswj@mail.sysu.edu.cn](mailto:ceswj@mail.sysu.edu.cn) (J. Wang)

**Characterization section**

The X-ray diffraction (XRD) spectrum of samples was recorded on a diffractometer (Rigaku D/MAX 2200 VPC) equipped with Cu Kα radiation (*λ* = 1.5405 Å) operated with 40 kV and 26 mA radiation. Room-temperature Raman spectrum was recorded using a micro-Raman system (Renishaw InVia, UK) with an excitation wavelength of 514.5 nm, with each sample tested in three different areas and yielding similar results. The morphology and elemental composition of the as-prepared sample were determined by scanning electron microscopy (SEM, FEI Quanta 200 Thermal FE environmental scanning electron microscopy) equipped with an energy-dispersive X-ray spectroscope (EDS) system. Diffuse reﬂectance spectra were recorded using a UV–vis–NIR spectrophotometer (Cary 5000, Varian) equipped with a double out-of-plane Littrow monochromator. Room-temperature Photoluminescence (PL), Photoluminescence excitation (PLE), Persistent luminescence spectra, and decay curves were measured with a high-resolution spectrofluorometer (Edinburgh Instruments, FLS1000) equipped with a 450 W xenon (Xe) lamp. Both 2D and 3D Thermoluminescence (TL) glow curves were collected in an LTTL-3DS thermoluminescence spectrophotometer (Guangzhou Radiation Technology Co., Ltd.) in the temperature range from RT to 700 K. Before 2D-TL measuring, the samples were exposed to a Hg lamp light source and then heated at a linear heating rate of 1 K·s^-1^. Before 3D-TL measuring, the samples were exposed to monochromatic light (260, 340, and 400 nm) from a 450 W Xe lamp and then heated at a linear heating rate of 1 K·s^-1^. The TL test was conducted in a darkroom under faint red light. Before the formal testing, the samples underwent a high-temperature treatment at 700 K to clear stored energy. The EPR spectra were recorded on an EPR spectrometer (Bruker A300) operating in the X-band frequencies (≈ 9.447 GHz) at the RT, with a microwave power of 2.12 mW. During the EPR experiment, the sample was excited in situ in the EPR cavity with the UV lamp (254 and 365 nm). Various UV light sources were employed for excitation-wavelength-dependent PersL testing. These included a 254 nm hand-held light (16 W), a 265 nm LED matrix light (100 W), a 305 nm LED matrix light (100 W), a 365 nm hand-held light (16 W), a 380 nm LED matrix light (50 W), and a 980 nm LED matrix light (30 W). The remaining UV light was provided by a 450 W xenon lamp on the spectrometer FLS1000.

**Analysis of carrier migration:** The trap depth and carrier densities in the traps were analyzed from 2D-TL spectra based on a classical multi-peak fitting method^1^:

$I\left( T \right)=n_{0}S\exp\left( -\frac{E}{kT} \right)\left[ (b-1)(S/\beta)\int_{T_{0}}^{T} \exp\left( -\frac{E}{kT} \right)dT+1 \right]$ (Equation S1)

where *E* is the trap depth (activation energy), *n_0_* is the concentration of trapped carriers, *k* is the Boltzmann constant, *b* is the kinetics order parameter, and β is the heating rate (1 K·s^-1^ for our experiment), *s* is the frequency factor. All these data were obtained using the native software mounted on the instrument.

**Calculations section**

**Parameter setting:** The intrinsic defects containing Bi^3+^-doped CaGa_2_O_4_ were modeled using a supercell (112 atoms) with new base-vectors of $\boldsymbol{a}$, $\boldsymbol{b}+\boldsymbol{c,}$ and $\boldsymbol{b}-\boldsymbol{c}$. The structural relaxation was carried out with density functional theory (DFT) calculations utilizing the Perdew−Burke−Ernzerof (PBE) exchange-correlation functional,^2^ as implemented in the Vienna *ab initio* Simulation Package (VASP).^3^ The cutoff energy was set to 520 eV and a single *k* point $\Gamma$ was employed. Semicore electrons are explicitly treated for Ca (3s^2^3p^6^4s^2^), Ga (3d^10^4s^2^4p^1^), O (2s^2^2p^4^), and Bi (5d^10^6s^2^6p^3^) with the projector augment wave (PAW) pseudopotentials in the relaxation and system energy calculations,^4^ and the spin-orbit coupling is included in Bi^3+^ ions. Electronic energy minimization is performed with a tolerance of ${10}^{-5}$ eV, while the force on each atom converges within 0.05 eV·Å^-1^. For improving the calculations of band gaps and providing a more accurate description of defect levels, the hybrid DFT of Heyd-Scuseria-Ernzerhof (HSE06)^5^ was utilized with an energy cutoff of 400 eV.

**Parameter setting:** The formation energy of a defect *X* in the charge state of *q* can be derived as follows:^6^

$E^{f}(X^{q},E_{F})=E_{\mathrm{tot}}\left[ X^{q} \right]-E_{\mathrm{tot}}\left[ \mathrm{bulk} \right]-\sum_{i} n_{i}\mu_{i}+qE_{F}$ (Equation S2)

where $E_{\mathrm{tot}}$ is the total energy of the optimized supercells, $n_{i}$are the numbers of the atoms of elements *i* which are added to ($n_{i}$ > 0) and or removed from ($n_{i}$ < 0) the perfect supercell, and $\mu_{i}$ are corresponding chemical potentials of these species. The Fermi energy level$E_{F}$ represents the chemical potential of the electrons in the host. The thermodynamic charge transition level $\epsilon(q_{1}/q_{2})$was utilized to predict the positions of defect levels, and it is defined as the Fermi level at which the defect formation energies of $X^{q_{1}}$ and $X^{q_{2}}$ equal each other. It can be deduced from **Equation S2** as:

$\epsilon(q_{1}/q_{2})=\frac{E^{f}\left( X^{q_{1}}, E_{f}=0 \right)-E^{f}\left( X^{q_{2}}, E_{f}=0 \right)}{q_{2}-q_{1}}$ (Equation S3)

where post hot corrections to the total energy of charged defects are employed following the method proposed in Ref 7.^7^ Considering the phosphor was prepared in air and Ga-poor environment, the atomic chemical potential $\mu_{i}$ of the element are provided as follows,

$\mu_{O}=\mu_{O_{2}(\mathrm{gas})}+\Delta\mu_{O}$ (Equation S4)

$\mu_{\mathrm{Ca}}=\mu_{CaO(bulk)}-\mu_{O}$ (Equation S5)

$\mu_{\mathrm{Bi}}=\frac{\mu_{\mathrm{Bi}_{2}O_{3}(\mathrm{bulk})}-{3\mu}_{O}}{2}$ (Equation S6)

where $\mu_{O_{2}(\mathrm{gas})}$, $\mu_{CaO(bulk)}$ and $\mu_{\mathrm{Bi}_{2}O_{3}(bulk)}$ are the calculated total energy per formula unit for the O_2_, CaO, and Bi_2_O_3_, respectively, and the $\Delta\mu_{O}=-0.9 \mathrm{eV}$ is estimated for the oxygen partial pressure (0.2 of the atmosphere pressure). The atomic chemical potential of Ga is determined by the constraints of $\mu_{\mathrm{CaG}a_{2}O_{4}(bulk)}=\mu_{\mathrm{Ca}}+2\mu_{\mathrm{Ga}}+4\mu_{O}$.

**Geometric structure and transition energies:** Generally, the most significant emission related to isolated Bi^3+^ in crystals originates from the equilibrium structure of the lowest one among the three types of excited states, *i*.*e.* MMCT state, CT state, and the *A* band (^3^P_0,1_) states. The equilibrium geometric structure of MMCT excited state was approximated as $\mathrm{CaG}a_{2}O_{4}:Bi^{4+}$, which was obtained by the geometric relaxation of the system with one electron removed from the $\mathrm{CaG}a_{2}O_{4}:Bi^{3+}$ supercell. Similarly, the equilibrium geometric structure of CT state was approximated by that of $\mathrm{CaG}a_{2}O_{4}:Bi^{2+}$. The ^3^P_0,1_ excited state was obtained by constraining the electron occupancy to (6s_1/2_)^1^(6p_1/2_)^1^ for $\mathrm{CaG}a_{2}O_{4}:Bi^{3+}$, where 6s_1/2_ and 6p_1/2_ are KS orbitals obtained with PBEsol by including SOC. The equilibrium geometric structures of the excited states of the Bi pairs were obtained via structural relaxation for spin triplets $2S+1=3$, which turned out to be Bi^2+^-Bi^4+^ type charge transfer states. Based on the Franck-Condon principle, the peak energy of excitation or emission for a given transition can be obtained approximately by the differences of the total energies of the excited and ground electronic state at the equilibrium geometric structure of the initial electronic states of the transition as mentioned in our previous work.^8^

**
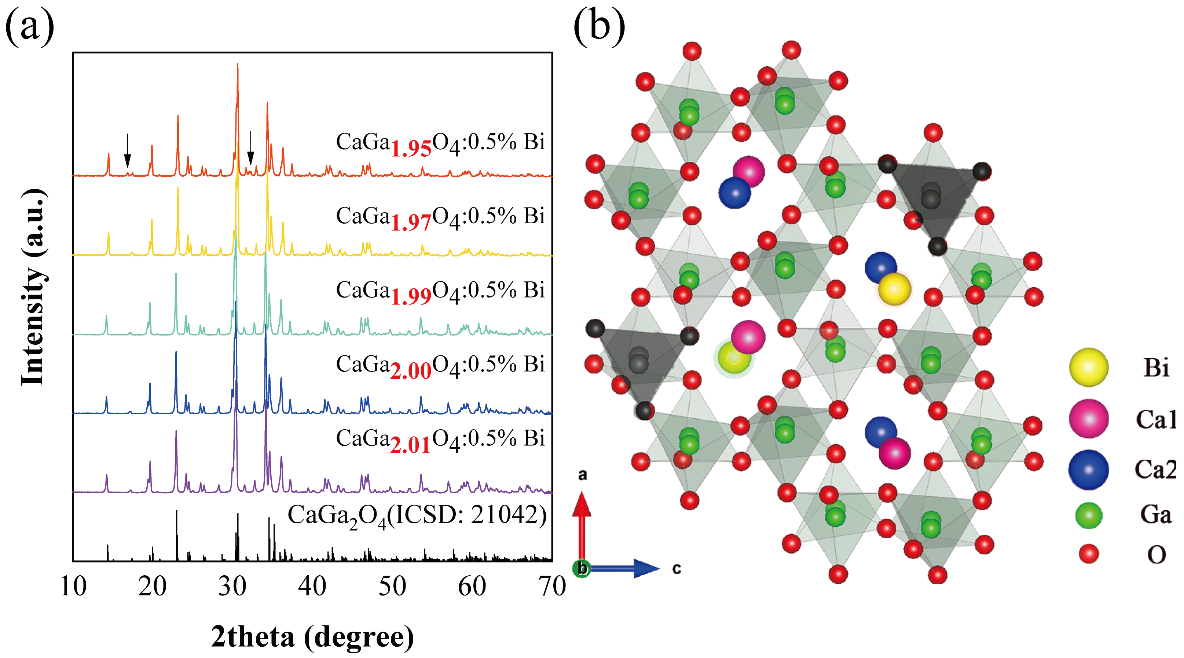
**

**Figure S1.** (a) XRD patterns of as-prepared CaGa_x_O_4_:0.5%Bi (*x* = 2.01, 2.00, 1.99, 1.97, and 1.95). The bottom curve shows the standard XRD pattern of CaGa_2_O_4_ which is rooted in the ICSD database. Two additional peaks are seen in the 2.5% Ga deficiency (*x* = 1.95) sample. The results suggest that the long-range ordered structure is maintained when the defect concentration is less than 2.5%. (b) Schematic diagram of the crystal structure of the Bi-doped nonstoichiometric sample, the shaded part is the missing [GaO_x_] structural unit. Note that there are 4 kinds of Ga sites and 8 kinds of O sites in the structure. For simplicity, we use only two colors for Ga and O.


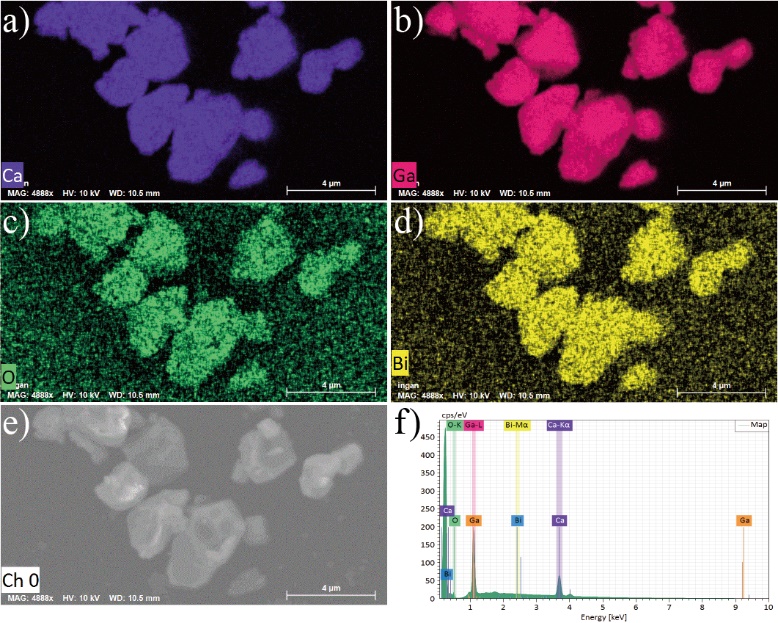


**Figure S2**. (a)-(d) EDS mapping of Ca, Ga, O, and Bi, (e) Electron micrograph showing the microstructure. (f) EDS analysis of GaGa_1_._97_O_4_:1%Bi. Note that to achieve a good signal-to-noise ratio for the Bi element, the nonstoichiometric sample (GaGa_1_._97_O_4_:1%Bi) with a Bi concentration of 1% was used.


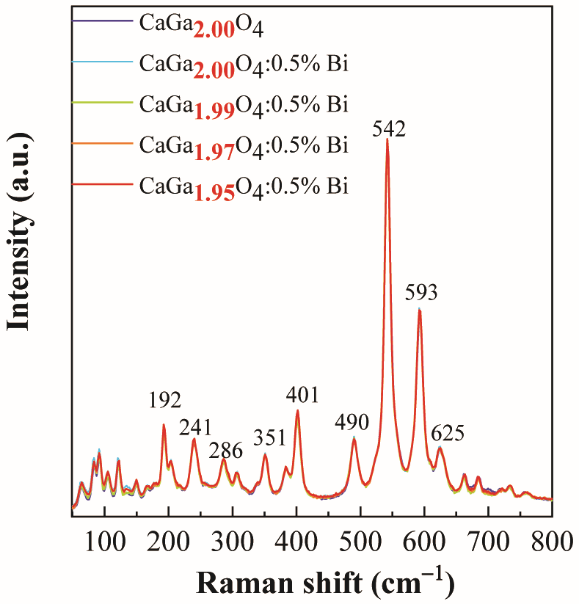


**Figure S3**. Normalized Raman spectra of CaGa_x_O_4_:0.5%Bi (*x* = 2.00, 1.99, 1.97, and 1.95) and undoped CaGa_2_O_4_.


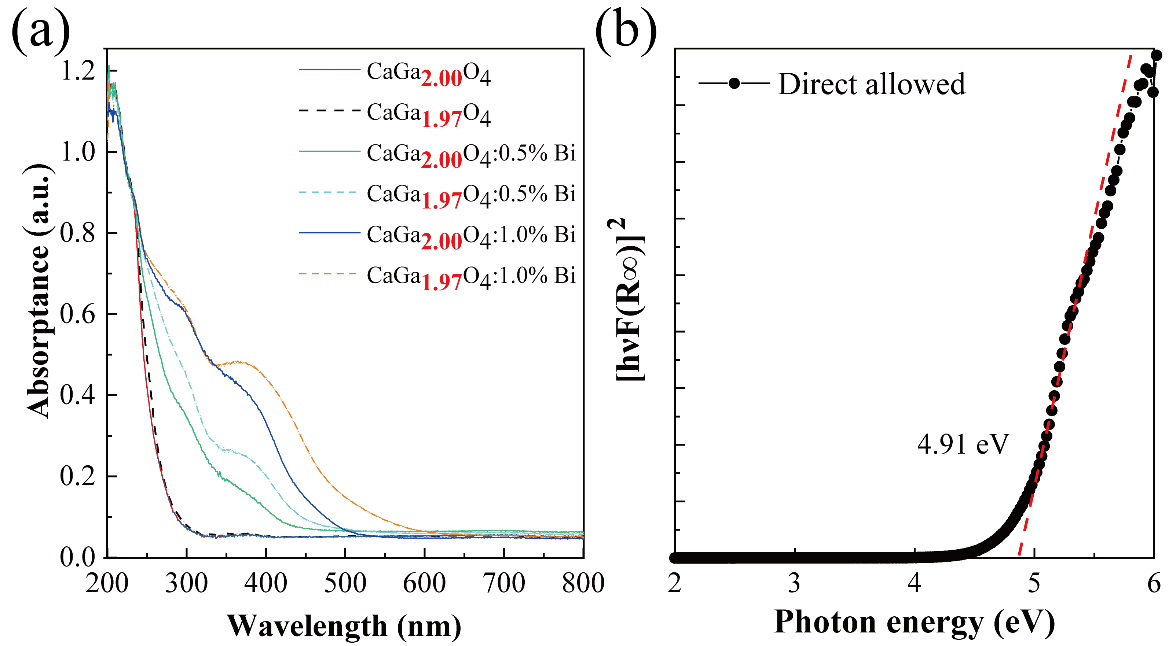


**Figure S4**. (a) UV-visible absorption spectra of undoped and Bi-doped CaGa_x_O_4_ (*x* = 2 and 1.97) samples. (b) Kubelka-Munk transformed reflectance spectra of CaGa_2_O_4_ for direct bandgap (Eg) measurements. By extrapolating the curve we obtain the direct optical bandgap at 4.91 eV.^9^ Comparing undoped CaGa_2_O_4_ and CaGa_1.97_O_4_, it can be seen that the V_Ga_ has no light absorption activity. But for Bi^3+^-doped CaGa_2_O_4_ and CaGa_1.97_O_4_ samples, the absorption properties of Bi^3+^ are significantly affected by V_Ga_. The above results indicate that V_Ga_ and the symbiotic defects may enhance the absorption of Bi^3+^ ions.


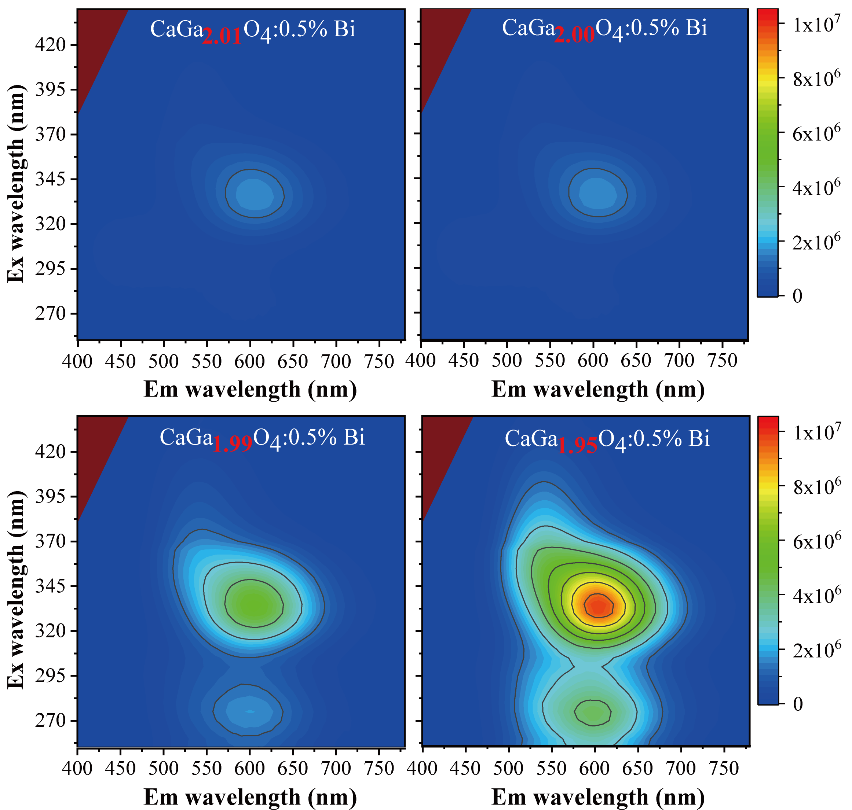


**Figure S5**. Two-dimensional excitation-emission graphs of the CaGa_x_O_4_:0.5%Bi (x=1.95, 1.99, 2.00, 2.01) samples. The graphs unambiguously revealed that all phases bear two classes of active centers with emission bands at circa *λ*_em_ = 605 and 542 nm, corresponding to excitation at about *λ*_ex_ = 335 and 355 nm for CaGa_x_O_4_:0.5%Bi (*x* = 2, 2.01), and *λ*_ex_ = 275/335 and 275/355 nm for CaGa_x_O_4_:0.5%Bi (*x* = 1.95-1.99) respectively.


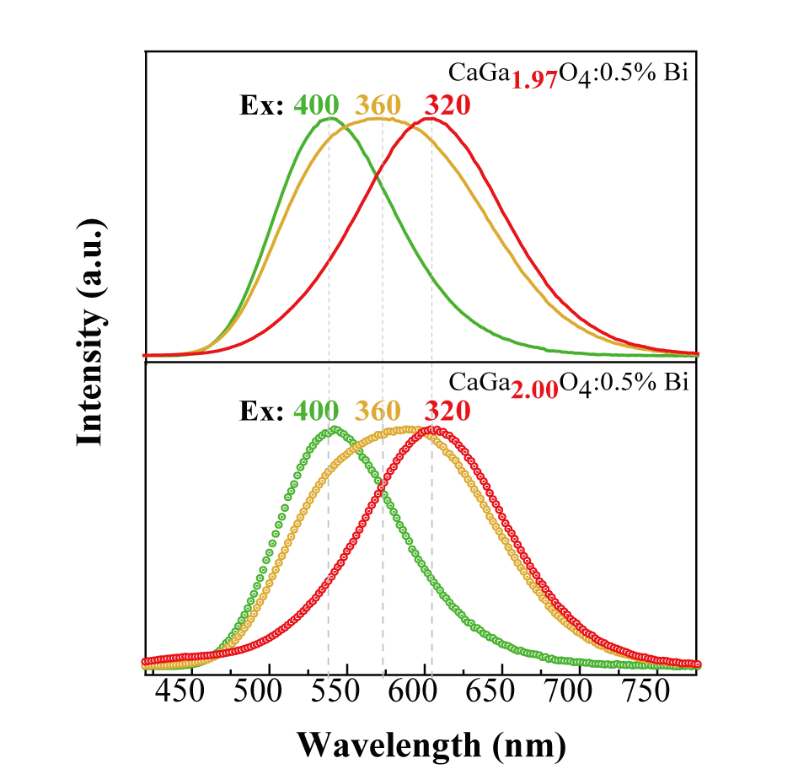


**Figure S6**. Normalized Excitation-wavelength-dependent emission spectra of CaGa_1.97_O_4_:0.5%Bi and CaGa_2_O_4_:0.5%Bi under excitation wavelengths of 320/360/400 nm.


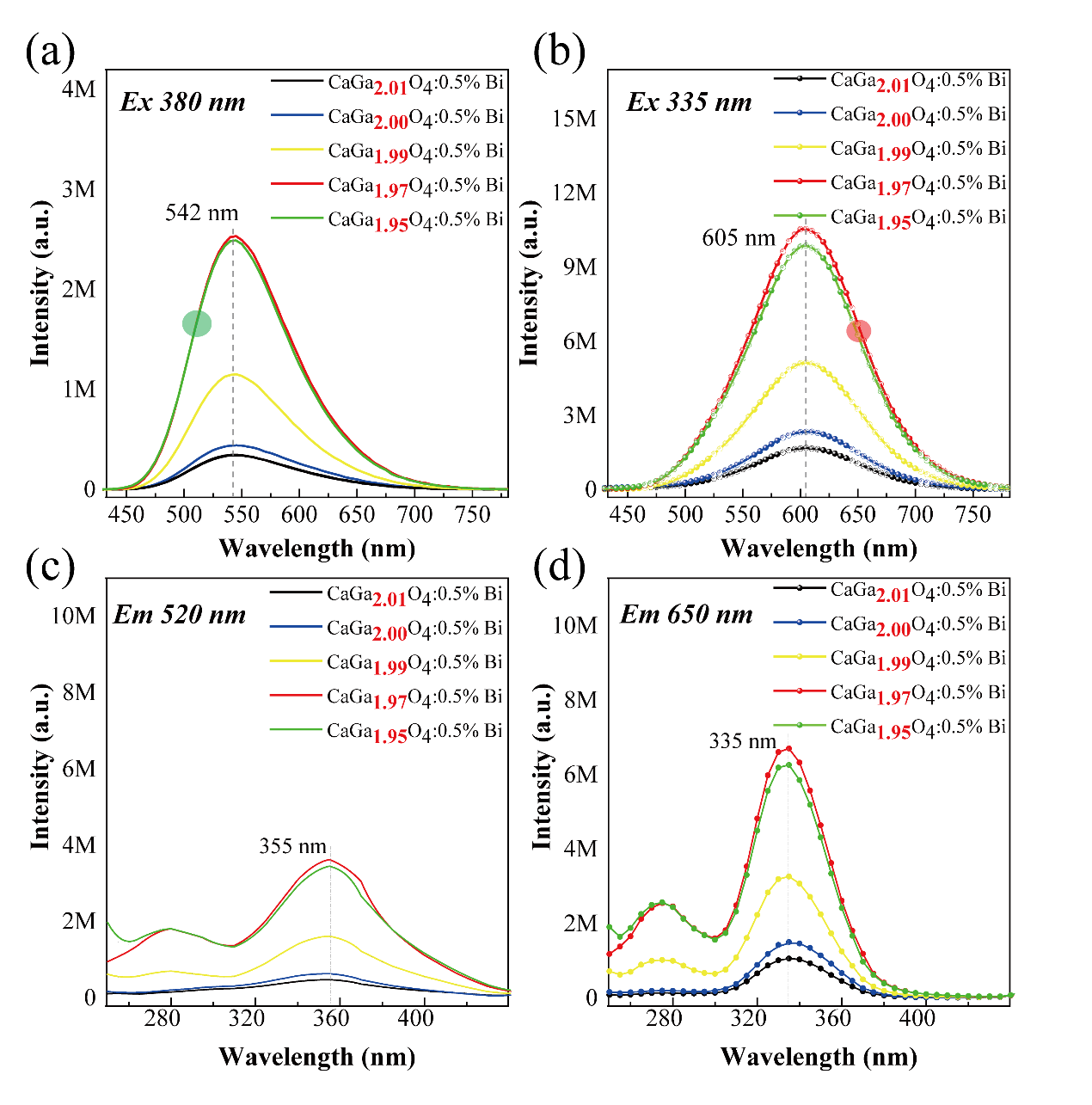


**Figure S7**. (a), (b) PL spectra of CaGa_x_O_4_:0.5%Bi (*x* = 1.95-2.01) excitation at *λ*_ex_=324 and 371 nm, respectively. (c), (d) PLE spectra of CaGa_x_O_4_:0.5%Bi (*x* = 1.95-2.01) emission at *λ*_em_=520 and 650 nm, respectively. Because the excitation and emission spectra of the two kinds of Bi^3+^ partially overlap, we did not detect the spectra at the peak value. For nonstoichiometric samples, the generation of the new excitation band around 275 nm in PLE spectra implies that there are some differences in the local environments of Bi^3+^ emitters.


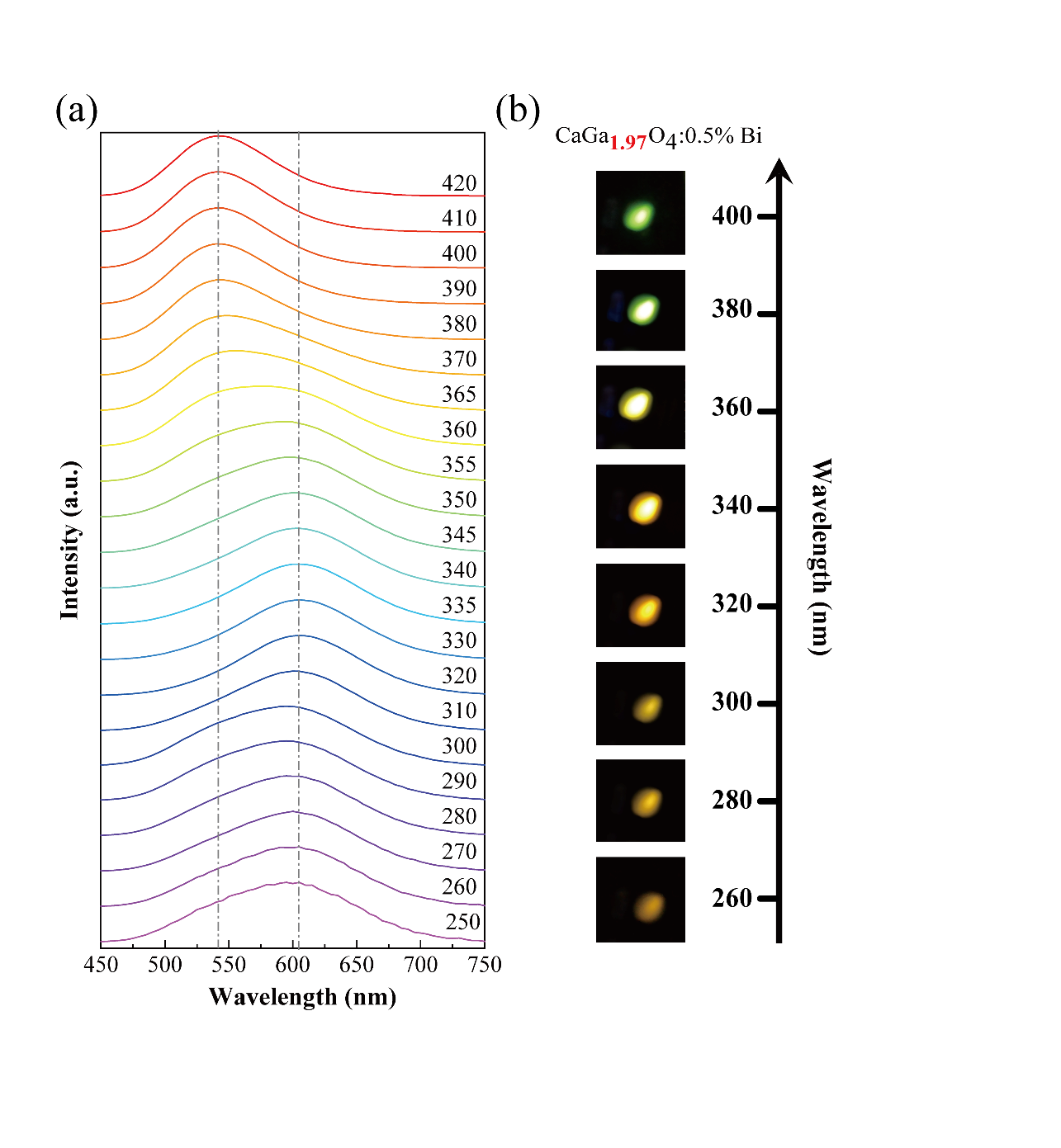


**Figure S8**. (a) Normalized steady-state PL spectra of CaGa_197_O_4_:0.5%Bi powder upon excitation from 250 to 420 nm under ambient conditions. (b) Corresponding photographs of the crystalline powder CaGa_197_O_4_:0.5%Bi under the UV light from 260 to 400 nm. Note that the emission wavelength is almost unchanged along with a variation of the excitation wavelength from 240 to 320 nm.


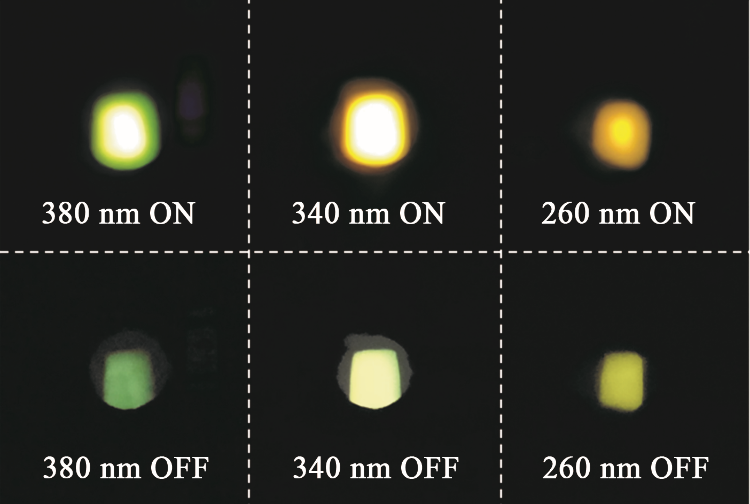


**Figure S9**. The steady-state PL and PersL images of the CaGa_1.97_O_4_:0.5%Bi sample with 380, 340, and 260 nm excitation by a xenon lamp. Note, the PersL images were taken at 30 s intervals for 3 minutes, with a spot size of approximately 1*1 cm.


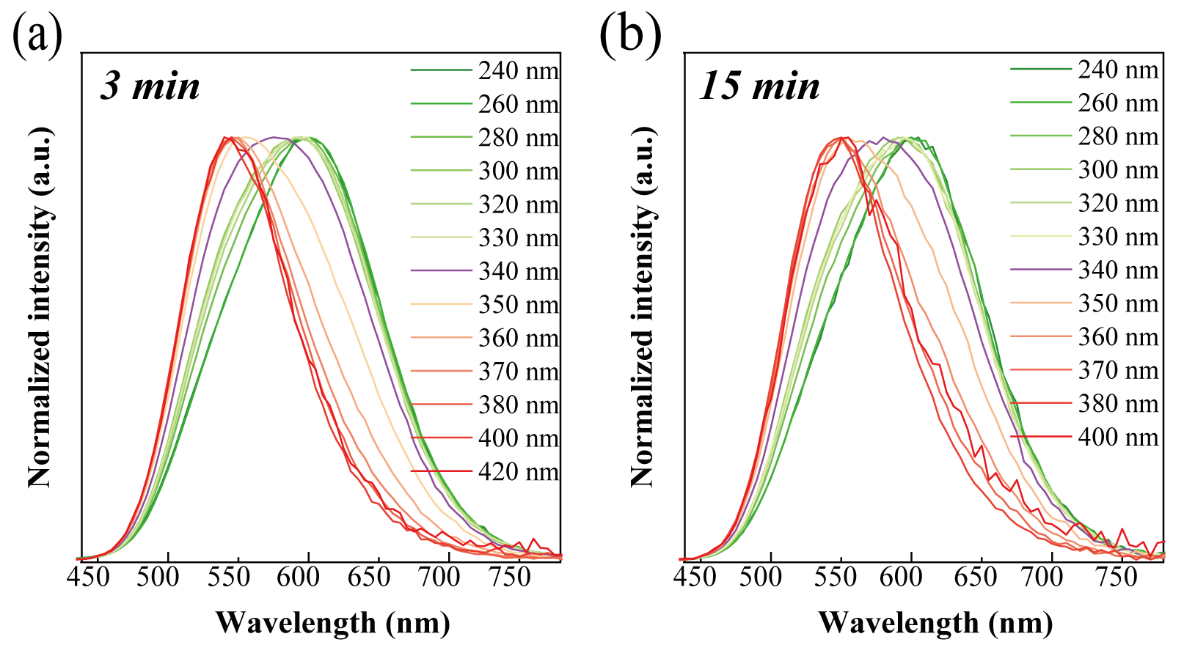


**Figure S10**. Normalized excitation-wavelength-dependent PersL spectra of CaGa_1_._97_O_4_:0.5%Bi at (a) 3 min and (b) 15 min after stopping excitation of specific UV light from Xe lamp for 5 min.


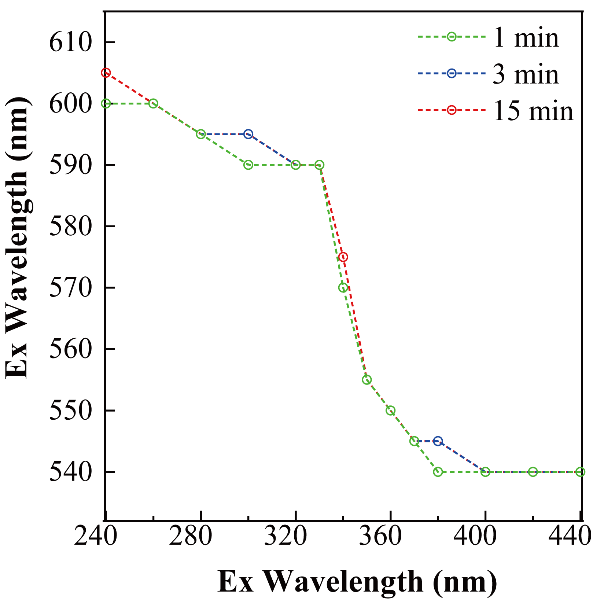


**Figure S11**. The corresponding persistent emission wavelength relations of various excitation wavelengths (240-440 nm). Note that the data were obtained from **Figure 2a, S10**. Notably, the persistent emission wavelength remains almost unchanged within 15 minutes, which indicates that the multicolor PersL can also be sustained.


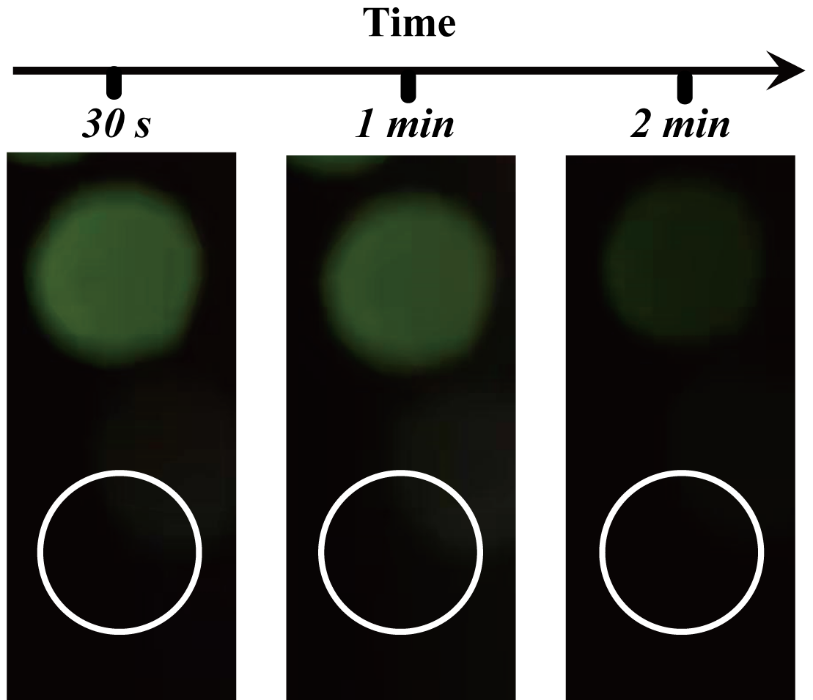


**Figure S12**. Images of CaGa_1_._97_O_4_:0.5%Bi (upper) and CaGa_2_O_4_:0.5%Bi (bottom) were taken at different PersL times (30 s min to 2 min) after irradiation by direct sunlight for 10 min. Imaging parameters: HUAWEI P20, ISO400/2 s.


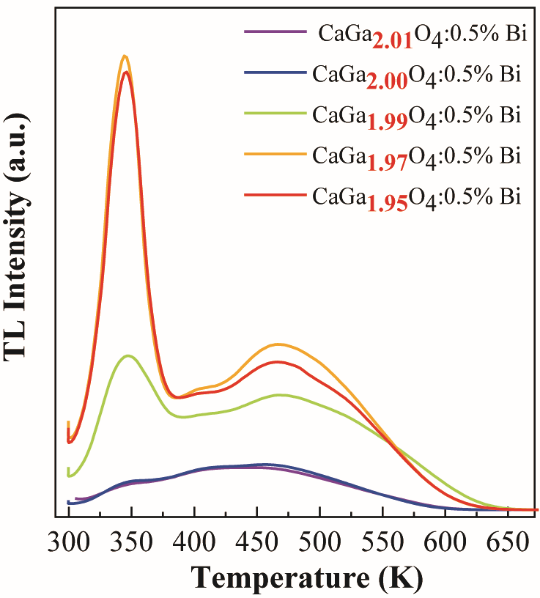


**Figure S13**. TL glow curves of CaGa_x_O_4_:0.5%Bi (*x* = 1.95-2.01) samples after irradiated by Hg lamp for 5 min. Note that a Hg lamp that can emit light from the deep UV to visible light regions is used as a light source to fill all traps.

The noticeable difference between the stoichiometric and Ga-deficient samples suggests that the traps are related to the V_Ga_ or symbiotic defects. As for stoichiometric CaGa_2_O_4_:0.5%Bi, the traps may come from a small amount of V_Ga_ or symbiotic defects introduced by Bi^3+^ substitution or a high-temperature sintering process. In addition, there are four kinds of Ga sites and eight kinds of O sites in the structure. As a consequence, multiple Ga vacancies and symbiotic defects (Ca_Ga_, Ga_Ca_, V_O_) may lead to multiple traps. Moreover, it is well known that shallow traps are easily emptied, and shallow traps at low temperatures contribute the most energy to afterglow emission. Notably, the TL intensity of CaGa_2_O_4_:0.5%Bi is only 4% of CaGa_1_._97_O_4_:0.5%Bi at 300 K, which explains that the PersL of the non-stoichiometric sample is much stronger than that of the stoichiometric sample.


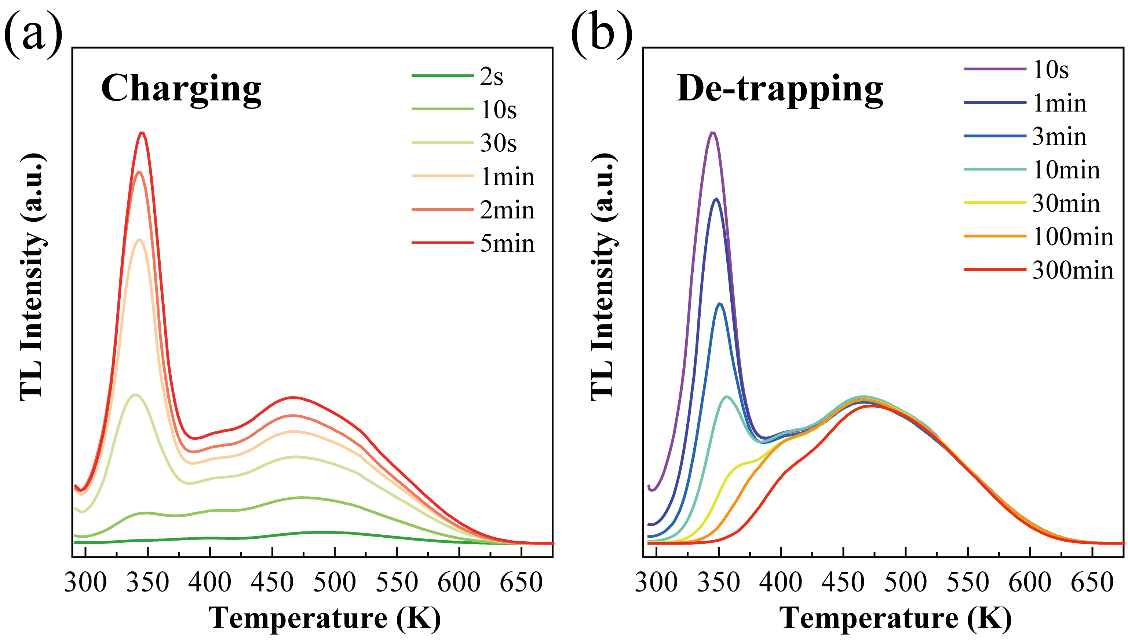


**Figure S14**. (a) TL curves of CaGa_1_._97_O_4_:0.5%Bi measured at 20 s after stopping the irradiation. The sample was pre-irradiated by an Hg lamp for different times (2 s - 5 min). (b) TL curves of CaGa_1_._97_O_4_:0.5%Bi acquired at various delay durations (10 s - 300 min) after irradiated by Hg lamp for 5 min.

**Figure S15**. The PBE calculated formation energies of the intrinsic defects and Bi^3+^ dopants in CaGa_2_O_4_ in different charge states as a function of the Fermi level, where the Fermi level is from 0 eV (VBM) to 2.96 eV (CBM).


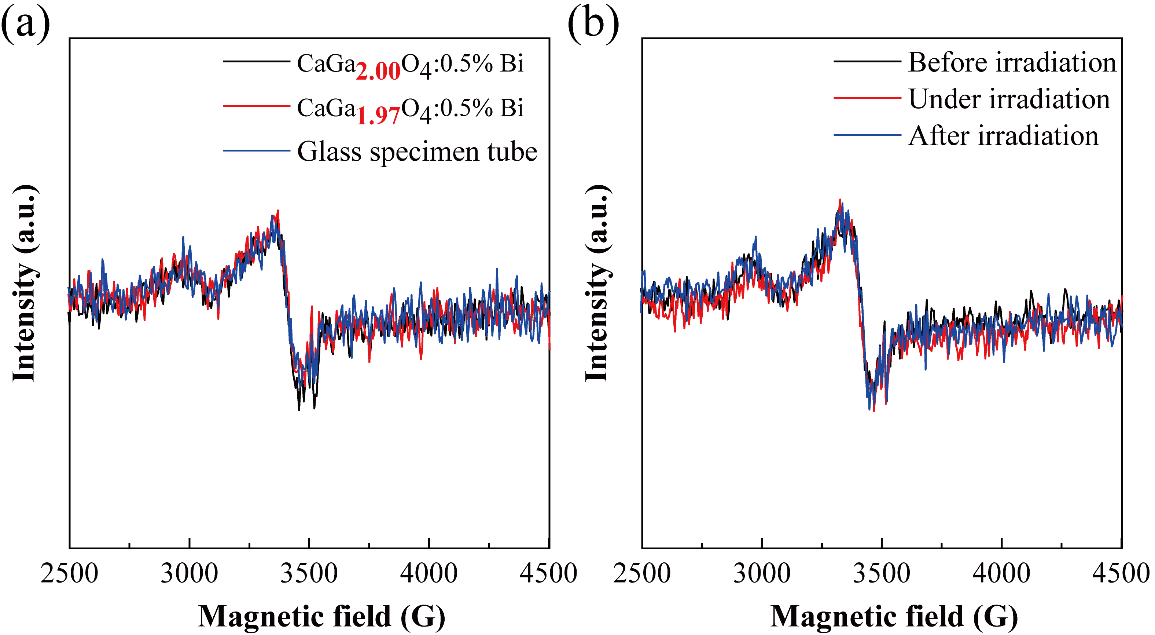


**Figure S16**. (a) EPR spectra of the CaGa_2_O_4_:0.5%Bi and CaGa_1_._97_O4:0.5%Bi samples, all the signals come from the impure ions, such as Fe or Cr in the glass specimen tube. (b) EPR spectra of CaGa_1_._97_O_4_:0.5%Bi measured at RT before, under, and after UV light irradiation for 5 min. Note, before, after, and in situ irradiating the samples with 254 and 365 nm light (inside the EPR cavity), we observed that all the EPR spectrum is perfectly superimposed to each other. Therefore, no evidence of electron or hole release from Bi^3+^ or trap center is shown here.


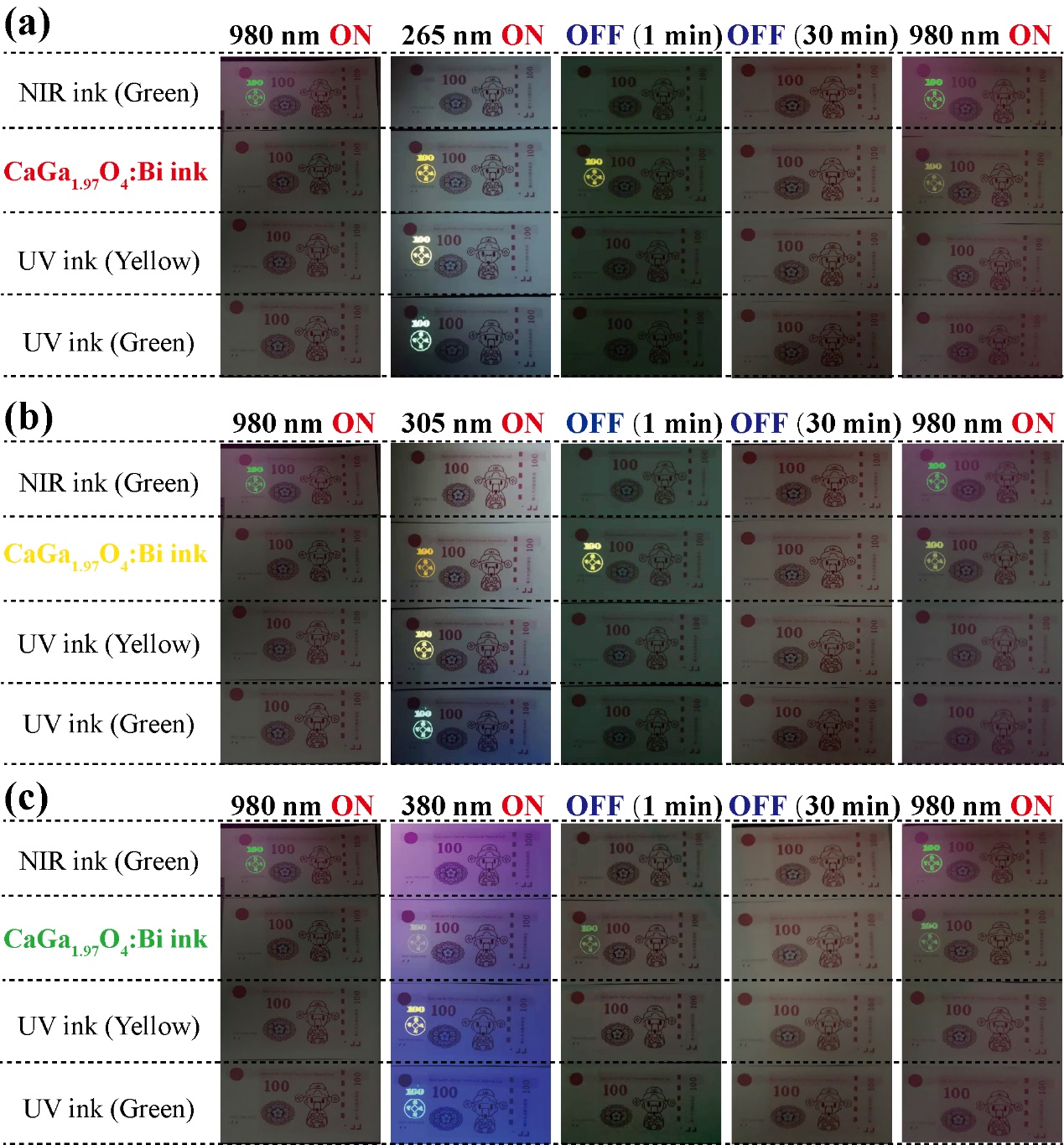


Figure S17. Images of the fake money patterned by CaGa_1_._97_O_4_:0.5%Bi ink under 980 nm light, (a) 265, (b) 305, (c) 380 nm LEDs ON and OFF with different times. For comparison, the invisible NIR ink (Green), UV ink (Yellow), and UV ink (Green) were also used to print the security pattern. To see the fluorescent image, we need to put the security pattern under the appropriate NIR or UV light, and only the steady-state luminescence with a single color can be seen.

**Table S1.** Fluorescence and structural parameters of two types of Bi^3+^ pairs luminescence center. Note, that doping with Bi^3+^ ion (1.03 Å, CN6) is expected to replace the Ca^2+^ site (1.00 Å, CN6), as the ionic radius of Ga^3+^ (0.62 Å, CN6) is very small. If we assume that the maximum Bi–O distance is less than 3.0 Å, the Bi coordination number (CN) is 7 for both Ca1 and Ca2, and the distortion index is 0.086 and 0.077 for $\mathrm{Bi}_{Ca1}$ (Bi in Ca1) and $\mathrm{Bi}_{Ca2}$ (Bi in Ca2), respectively. The value of the distortion index is obtained by VESTA software mathematic calculating. Meantime, the relatively short bond distance and large distortion index in the $\mathrm{Bi}_{Ca1}$ site probably results in relatively higher transition levels and a large nephelauxetic effect. Furthermore, from the theoretic and practical study, we assign the orange emission to the A band excitation of $\mathrm{Bi}_{Ca1}^{3+}$ and that of the green emission to the A band excitation of $\mathrm{Bi}_{Ca2}^{3+}$.

| Ground states of  Bi pairs | Excited states of  Bi pairs | Excitation  (nm) | Emission  (nm) | Stokes shift (cm^−1^) | Average bond length (Å) |
| --- | --- | --- | --- | --- | --- |
| $\mathrm{Bi}_{Ca1}^{3+}-\mathrm{Bi}_{Ca2}^{3+}$ | $\mathrm{Bi}_{Ca1}^{4+}-\mathrm{Bi}_{Ca2}^{2+}$ | 335 | 605 | 13322 | 2.47 |
| $\mathrm{Bi}_{Ca1}^{3+}-\mathrm{Bi}_{Ca2}^{3+}$ | $\mathrm{Bi}_{Ca1}^{2+}-\mathrm{Bi}_{Ca2}^{4+}$ | 355 | 542 | 9719 | 2.53 |

**Table S2.** Trapping parameters were obtained from the TL glow curves of the CaGa_1_._97_O_4_:0.5%Bi^3+^ samples with different [excitation](javascript:;) [wavelength](javascript:;)s.

| [Excitation](javascript:;)  [wavelength](javascript:;) (nm) | Peak | Peak position (K) | Order of kinetics | Activation energy (eV) | Frequency factor (s^-1^) | Trap density (a. u.) |
| --- | --- | --- | --- | --- | --- | --- |
| 260 nm | A | 351 | 2 | 0.76 | 3.09 x 10^10^ | 1.68 x 10^6^ |
|  | B | 405 | 2 | 0.87 | 1.85 x 10^10^ | 1.14 x 10^6^ |
|  | C | 454 | 2 | 1.01 | 3.90 x 10^10^ | 1.43 x 10^6^ |
|  | D | 499 | 2 | 1.12 | 5.24 x 10^10^ | 1.32 x 10^6^ |
|  | E | 545 | 2 | 1.21 | 3.85 x 10^10^ | 8.67 x 10^5^ |
| 340 nm | A | 354 | 2 | 0.74 | 1.23 x 10^10^ | 2.02 x 10^6^ |
|  | B | 402 | 2 | 0.86 | 1.51 x 10^10^ | 2.09 x 10^6^ |
|  | C | 450 | 2 | 1.00 | 4.41 x 10^10^ | 2.30 x 10^6^ |
|  | D | 495 | 2 | 1.10 | 3.61 x 10^10^ | 2.46 x 10^6^ |
|  | E | 543 | 2 | 1.22 | 6.44 x 10^10^ | 1.68 x 10^6^ |
| 400 nm | A | - | - | - | - | - |
|  | B | 393 | 2 | 0.85 | 1.64 x 10^10^ | 7.78 x 10^5^ |
|  | C | 440 | 2 | 1.01 | 5.96 x 10^10^ | 9.80 x 10^5^ |
|  | D | 489 | 2 | 1.11 | 6.08 x 10^10^ | 1.91 x 10^6^ |
|  | E | 533 | 2 | 1.24 | 1.34 x 10^11^ | 1.49 x 10^6^ |

**Reference**

1. Bos AJJ. Theory of thermoluminescence. *Radiat. Meas.* **41**, 45-56 (2006).

2. Perdew JP, Burke K, Ernzerhof M. Generalized gradient approximation made simple. *Phys. Rev. Lett.* **77**, 3865 (1996).

3. Kresse G, Furthmüller J. Efficient iterative schemes for ab initio total-energy calculations using a plane-wave basis set. *Phys. Rev. B* **54**, 11169 (1996).

4. Kresse G, Joubert D. From ultrasoft pseudopotentials to the projector augmented-wave method. *Phys. Rev. B* **59**, 1758 (1999).

5. Heyd J, Scuseria GE, Ernzerhof M. Hybrid functionals based on a screened Coulomb potential. *J. Chem. Phys.* **118**, 8207-8215 (2003).

6. Freysoldt C*, et al.* First-principles calculations for point defects in solids. *Rev. Mod. Phys.* **86**, 253 (2014).

7. Durrant T, Murphy S, Watkins M, Shluger A. Relation between image charge and potential alignment corrections for charged defects in periodic boundary conditions. *J. Chem. Phys.* **149**, (2018).

8. Lou B, Wen J, Ning L, Yin M, Ma C-G, Duan C-K. Understanding the defect levels and photoluminescence in a series of bismuth-doped perovskite oxides: First-principles study. *Phys. Rev. B* **104**, 115101 (2021).

9. Cai X*, et al.* An investigation about the luminescence mechanism of SrGa_2_O_4_: Eu^3+^ showing no detectable energy transfer from the host to the dopant ions. *J. Lumin.* **200**, 169-174 (2018).
