# Supplementary material for: Excitation-wavelength-dependent persistent luminescence from single-component nonstoichiometric CaGaxO4:Bi for dynamic anti-counterfeiting
Source: Light Sci Appl. 2024 Oct 10;13:286. doi: 10.1038/s41377-024-01635-7 (PMC11467341; doi:10.1038/s41377-024-01635-7)
Supplement: Supplementary file 2 — Annotation for movie 1–2 [file 41377_2024_1635_MOESM2_ESM.docx]

[**Supplementary Movie**](https://static-content.springer.com/esm/art%3A10.1038%2Fncomms9001/MediaObjects/41467_2015_BFncomms9001_MOESM915_ESM.mov) **1**

This movie depicts excitation-wavelength-dependent PersL exhibited by the CaGa_1_._97_O_4_:0.5%Bi embedded label, with excitation wavelengths selected at 265, 305, 365, and 380 nm. Meanwhile, commercial yellow and green ink can only exhibit single emissions. Note, the high-power red light LED is used to clear the stored PersL energy.

[**Supplementary Movie**](https://static-content.springer.com/esm/art%3A10.1038%2Fncomms9001/MediaObjects/41467_2015_BFncomms9001_MOESM915_ESM.mov) **2**

This movie depicts color-turning PSL demonstrated by the CaGa_1_._97_O_4_:0.5%Bi embedded label. In contrast, commercial yellow and green ink can only exhibit single emissions. Additionally, the 980 nm LED is employed to release the stored multicolor PersL energy, while the high-power red light LED is used to clear the stored PersL energy.
